# Visualizing BDNF Transcript Usage During Sound-Induced Memory Linked Plasticity

**DOI:** 10.3389/fnmol.2018.00260

**Published:** 2018-07-31

**Authors:** Lucas Matt, Philipp Eckert, Rama Panford-Walsh, Hyun-Soon Geisler, Anne E. Bausch, Marie Manthey, Nicolas I. C. Müller, Csaba Harasztosi, Karin Rohbock, Peter Ruth, Eckhard Friauf, Thomas Ott, Ulrike Zimmermann, Lukas Rüttiger, Thomas Schimmang, Marlies Knipper, Wibke Singer

**Affiliations:** ^1^Department of Pharmacology, Institute of Pharmacy, Toxicology, and Clinical Pharmacy, University of Tübingen, Tübingen, Germany; ^2^Department of Otolaryngology, Tübingen Hearing Research Centre, Molecular Physiology of Hearing, University of Tübingen, Tübingen, Germany; ^3^Animal Physiology Group, Department of Biology, University of Kaiserslautern, Kaiserslautern, Germany; ^4^Section of Physiological Acoustics and Communication, Department of Otolaryngology, Tübingen Hearing Research Center, University of Tübingen, Tübingen, Germany; ^5^Transgenic Facility Tübingen, University of Tübingen, Tübingen, Germany; ^6^Instituto de Biologíay Genética Molecular, Universidad de Valladolid, Consejo Superior de Investigaciones Científicas (CSIC), Valladolid, Spain

**Keywords:** *Bdnf* exon-IV, *Bdnf* exon-VI, LTP, memory acquisition, feed-forward inhibition, parvalbumin, vasculature, sound-accentuation

## Abstract

Activity-dependent BDNF (brain-derived neurotrophic factor) expression is hypothesized to be a cue for the context-specificity of memory formation. So far, activity-dependent BDNF cannot be explicitly monitored independently of basal BDNF levels. We used the BLEV (***B***DNF-***l****ive-****e****xon*-***v****isualization*) reporter mouse to specifically detect activity-dependent usage of *Bdnf* exon-IV and -VI promoters through bi-cistronic co-expression of CFP and YFP, respectively. Enriching acoustic stimuli led to improved peripheral and central auditory brainstem responses, increased Schaffer collateral LTP, and enhanced performance in the Morris water maze. Within the brainstem, neuronal activity was increased and accompanied by a trend for higher expression levels of *Bdnf* exon-IV-CFP and exon-VI-YFP transcripts. In the hippocampus BDNF transcripts were clearly increased parallel to changes in parvalbumin expression and were localized to specific neurons and capillaries. Severe acoustic trauma, in contrast, elevated neither *Bdnf* transcript levels, nor auditory responses, parvalbumin or LTP. Together, this suggests that critical sensory input is essential for recruitment of activity-dependent auditory-specific BDNF expression that may shape network adaptation.

## Introduction

Brain-derived neurotrophic factor (BDNF), identified in 1982 (Barde et al., 1982), is recognized as key modulator of synaptic plasticity during homeostatic readjustment processes and a master regulator of energy homeostasis (for review see: Bramham and Messaoudi, 2005; Rauskolb et al., 2010; Park and Poo, 2013; Marosi and Mattson, 2014; Nahmani and Turrigiano, 2014; Jeanneteau and Arango-Lievano, 2016; Mitre et al., 2017). BDNF is well-known for its involvement in Schaffer collateral long-term potentiation (LTP) (Minichiello, 2009) as well as in inhibition (Huang and Reichardt, 2001; Wardle and Poo, 2003; Lu et al., 2009; Waterhouse and Xu, 2009; Duguid et al., 2012; Park and Poo, 2013; Parkhurst et al., 2013; Nahmani and Turrigiano, 2014). Nevertheless, we do not yet understand BDNF's influence on circuit stabilization in the adult system or its function in platelets (Chacón-Fernández et al., 2016), capillary endothelial cells (Donovan et al., 2000), microglia, and astrocytes (Ferrini and De Koninck, 2013; Parkhurst et al., 2013) during homeostatic readjustment processes (Nahmani and Turrigiano, 2014). Among other reasons, this is due to the difficulty of detecting the very low (Dieni et al., 2012) endogenous expression of BDNF in the mature central nervous system (CNS) (Dieni et al., 2012). Furthermore, many BDNF mutant mouse lines present severe phenotypes that complicate analysis of BDNF under normal physiological conditions (Ernfors et al., 1994; Rios et al., 2001; Postigo et al., 2002; Chourbaji et al., 2008; Hong et al., 2008; Sakata et al., 2009; Rauskolb et al., 2010; Lyons and West, 2011; Zuccotti et al., 2012; Vanevski and Xu, 2013; Mallei et al., 2015; Hill et al., 2016; Maynard et al., 2016).

Part of the multifaceted functions of BDNF might be obfuscated by the complex structure of the *Bdnf* gene, which is comprised of eight independently transcribed non-coding exons (I-VIII), each of which is spliced to a common protein encoding exon (IX) (Timmusk et al., 1993; Aid et al., 2007), resulting in multiple transcripts that display different stability, targeting, and translatability (Vaghi et al., 2014). BDNF expression from each of these eight different promoters is independently regulated (Vaghi et al., 2014). Of particular interest are exon-IV and exon-VI, both containing promoters directly or indirectly regulated by neuronal activity (Hong et al., 2008; West et al., 2014; Tuvikene et al., 2016). Dysfunction of these two *Bdnf* transcripts is associated with deficits in sleep, fear, and memory (Hill et al., 2016), as well as depression (Sakata et al., 2010), cortical inhibition deficiency (Hong et al., 2008), and cognitive decline (Vaghi et al., 2014; Mallei et al., 2015). Moreover, downregulation of activity-dependent BDNF was observed in stress-related neuropsychiatric disorders (Pariante, 2009; Castrén and Rantamäki, 2010) or in states of increased glucocorticoid resistance, for example during chronic stress (Bath et al., 2013; Gray et al., 2013; Jeanneteau and Arango-Lievano, 2016). Any stress reaction that may result in behavioral changes related to external cues requires an activity-dependent signal to provide context specificity to the otherwise ubiquitous glucocorticoid receptor (GR)-mediated stress response (de Kloet, 2014). For example, metabolic support for behaviorally relevant motor learning can only be provided through activity-dependent reallocation of GR effects (Liston et al., 2013; Arango-Lievano et al., 2015; Jeanneteau and Arango-Lievano, 2016). In this context, BDNF was previously hypothesized to provide the corresponding signal (Jeanneteau and Arango-Lievano, 2016).

So far, however, it was technically impossible to detect increased activity-dependent BDNF expression above the background of basal BDNF levels. We have generated ***B***DNF-***l****ive-****e****xon-****v****isualization* (BLEV) knock-in reporter mice to specifically detect BDNF in response to *Bdnf* exon-IV and -VI promoter usage (Singer et al., submitted). The generation and validation of this new reporter mouse line is described in detail in Singer et al. (submitted). In BLEV mice, *Bdnf* exon-IV and -VI mRNA translation sites are tagged by bi-cistronic co-expression of cyan- and yellow-fluorescent-protein (CFP and YFP), respectively (Singer et al., submitted). BLEV reporter mice are viable without any BDNF-related mutant phenotype (Singer et al., submitted). Importantly, they allow the detection of *Bdnf* exon-IV-CFP and *Bdnf* exon-VI-YFP at sites of BDNF protein expression in neurons, microglia, astrocytes, and capillaries (Singer et al., submitted), cell types previously shown to express BDNF (Edelmann et al., 2014; Serra-Millàs, 2016). Furthermore, in BLEV mice it is possible to observe activity-dependent hippocampal BDNF expression in response to glutamate receptor activation through either CFP and YFP fluorescence or quantification of protein tags by Western blot (Singer et al., submitted). To test if BLEV mice also allow monitoring of activity-dependent BDNF during behaviorally relevant long-term adaptive processes in response to external cues, we monitored *Bdnf*-transcript changes in BLEV reporter mice following different sound exposure conditions. Previous experiments demonstrated that exposures to defined enriching, mild traumatic, or severe traumatic sound pressure levels (SPL) caused long-lasting alterations to sound-sensitivity and differentially induced hippocampal plasticity (Singer et al., 2013). Likewise, acoustically induced differences in central sound sensitivity correlated with sensitivity to stress as shown through a social stress paradigm or pharmacological inhibition of stress receptors (Singer et al., 2018).

In the present study we observed persistent sound sensitivity changes after these sound exposure paradigms in BLEV mice. In the auditory brainstem, these changes were accompanied by increased expression of VGLUT1, an established marker of excitation in auditory fibers, and a tendency to upregulate *Bdnf* exon-IV and exon-VI. These observations were specific for the auditory system and could not be detected in the olfactory bulb. Furthermore, different exposure levels correlated with altered *Bdnf* exon-IV and exon-VI expression in specific hippocampal neurons and vascular cells. In the hippocampus, this was paralleled by altered levels of GluA2 and parvalbumin expression and associated with a changed balance between excitatory vs. inhibitory inputs to CA1 pyramidal cells, underlined by altered Schaffer collateral LTP and spatial memory performance. These findings can only be explained by a critical sensory input that drives activity-dependent BDNF expression toward long-term adaptive responses.

## Methods

### Animals

Animal care and use and experimental protocols correspond to national and institutional guidelines and were reviewed and approved by the animal welfare commissioner and the regional board for animal experimentation. All experiments were performed according to the European Union Directive 2010/63/EU for the protection of animals used for experimental and other scientific purposes. Mice were kept according to national guidelines for animal care in an SPF animal facility at 25°C on a 12/12 h light/dark cycle with average noise levels of around 50–60 dB.

### Vector construct for a transgenic BDNF mouse

For a detailed description of the generation of the new mouse model please see Singer et al., (submitted). In brief, the *Bdnf* exon-IV and -VI sequence, both including the corresponding promoter sequences, were extended by CFP or YFP, respectively, both containing a stop codon. A HA-tag was added to *Bdnf* exon-IV-CFP and a cMyc-tag to *Bdnf* exon-VI-YFP. The translation of the protein-coding *Bdnf* exon-IX is enabled by an IRES sequence, which keeps the mRNA at the ribosome, despite the presence of a stop codon. Additionally, the growth-associated protein 43 (GAP43), is added to anchor the fluorescent proteins at the site of translation. This allows differential monitoring of the non-coding *Bdnf* exon-IV and *Bdnf* exon-VI by the fluorescent proteins CFP and YFP without interfering with *Bdnf* exon-IX.

### Hearing measurements and noise exposure

The hearing function of 2–3 months old BLEV reporter mice of both sexes was studied by measuring auditory brainstem responses (ABRs), as previously described (Zuccotti et al., 2012; Rüttiger et al., 2013). For noise exposure, animals were exposed to 10 kHz for 40 min at 80, 100, or 120 dB SPL while under anesthesia. For ABR recordings and noise exposure, we anesthetized the animals with an intraperitoneal injection of a mixture of ketamine-hydrochloride (75 mg/kg body weight, Ketavet, Pharmacia, Erlangen, Germany) and xylazine hydrochloride (5 mg/kg body weight, Rompun, Bayer, Leverkusen, Germany). Additional doses of anesthetics were administered if needed. Sham-exposed animals were anesthetized and placed in the reverberating chamber but not exposed to acoustic stimulus (i.e., the speaker remained turned off). Mice were randomly allocated to the different treatment groups.

### Tissue preparation

For protein isolation, brains were dissected with small forceps and immediately frozen in liquid nitrogen and stored at −80°C before use. Brain and cochlear tissue for immunohistochemistry was prepared as previously described (Singer et al., 2016).

### Protein isolation and western blot

For isolation of cMyc-tagged proteins and HA-tagged proteins, the Mild Purification kit and the HA-tagged Protein Purification kit were used, respectively (BiozolDiagnostica). In brief, tissues were dissolved in a lysis buffer (CelLytic M, Sigma-Aldrich) and incubated for 1 h with anti-cMyc or anti-HA tag beads suspension. The suspension was then centrifuged and washed; cMyc- and HA-tagged proteins were eluted with Elution Peptide Solution from the kit. All samples underwent the same procedures. The flow-through of the IP after extraction of the HA- and cMyc-tagged proteins was loaded on the gel to detect GAPDH (see Supplementary Table 1) and all other antibodies (expect of RCFP). Equal amounts of proteins are loaded for each lane.

Proteins were separated by electrophoresis and placed on a transfer membrane; non-specific epitopes of the membrane were blocked with 5% milk powder solution and incubated overnight at 4°C with the primary antibody, see Supplementary Table 1. Membranes for one approach were incubated with several primary antibodies at the same time, see originals in Supplementary Figures 2, 3. On the second day, the membrane was washed three times with Tris buffer/0.1% Tween 20; the secondary antibody (see Supplementary Table 1) was incubated for 1 h at room temperature in a sealed envelope. The membrane was washed again three times with Tris buffer/0.1% Tween 20. Proteins were visualized with ECL Prime WB Detection Reagent (GE Healthcare) using the Proxima 2700 (Isogen Life Science). For protein sizes, see Supplementary Table 1.

### Immunohistochemistry and ribbon counting

Cochleae were isolated, fixed, cryosectioned, and stained as described (Tan et al., 2007; Singer et al., 2016). Image acquisition and CtBP2/RIBEYE-immunopositive spot counting were carried out as previously described (Zuccotti et al., 2012; Singer et al., 2016).

Immunohistochemistry on brain sections was carried out as previously described (Singer et al., 2016). Antibodies are described in Supplementary Table 1.

### Field excitatory postsynaptic potential (fEPSP) recordings in hippocampal slices

Extracellular fEPSP recordings were performed according to standard methods as previously described (Matt et al., 2011; Chenaux et al., 2016).

In brief: 400 μm thick slices were cut on a vibratome (Leica VT 1000S) while submerged in ice-cold dissection buffer (composition in mM) 127 NaCl, 1.9 KCl, 1.2 KH_2_PO_4_, 26 NaHCO_3_, 10 D-glucose, 23 MgSO_4_, and 1.1 CaCl_2_, saturated with 5% CO_2_ and 95% O_2_ (pH 7.4). Slices were incubated in oxygenated artificial cerebrospinal fluid (ACSF, in mM: 127 NaCl, 26 NaHCO_3_, 1.2 KH_2_PO_4_, 1.9 KCl, 2.2 CaCl_2_, 1 MgSO_4_ and 10 D-glucose; pH 7.4) for 1 h at 30°C and then stored at room temperature. Recordings were performed in a submerged-type recording chamber (Warner Instruments). Stimulation (TM53CCINS, WPI) and recording electrodes (ACSF-filled glass pipettes, 2–3 MΩ) were positioned in the stratum radiatum to record Schaffer collateral fEPSPs. Signals were amplified with an Axopatch 200B (Molecular Devices), digitized at 5 kHz with an ITC-16 (HEKA) and recorded using WinWCP from the Strathclyde Electrophysiology Suite. Stimuli (100 μs) were delivered through a stimulus isolator (WPI). The same stimulus intensity was used during baseline recording (0.067 Hz) and induction of long-term potentiation (LTP) using 100 stimuli given at 100 Hz (1 s). The baseline was determined by the average of fEPSP initial slopes from the period before the tetanus. The level of LTP was determined by the average of fEPSP initial slopes from the period between 50 and 60 min after the tetanus. For wash-in experiments with 50 μM picrotoxin (Sigma), the level of potentiation was determined by the average of fEPSP initial slopes from the period between 15 and 20 min after the beginning of wash-in. Before tetanic stimulation or wash-in, each slice was used to record input-output relation (IOR) and paired-pulse facilitation (PPF) at the same stimulation strength as LTP recordings. Four traces were averaged for each data point.

### Morris water maze (MWM)

The Morris water maze test was performed as previously described (Bausch et al., 2015) using 3.4–4.5 and 1.7–2.7-month-old homozygous and heterozygous female BLEV reporter mice 10 days after exposure to sham or 80 dB SPL. We included as control group 11 homozygous and seven heterozygous mice (four males, 14 females) and as 80 dB SPL exposure group 10 homozygous and seven heterozygous mice (two males, 15 females). Age and sex was equally distributed among the groups. In mice, no significant difference is observed in MWM performance between males and females (Jonasson, 2005). Furthermore, the chosen age ranges are well below the age in which declining memory performance is to be expected (see e.g., de Fiebre et al., 2006). Therefore, we expected either sex or age differences to significantly influence variability.

The learning paradigm was specifically designed to demonstrate improved learning performance. We therefore opted for a very challenging task consisting of only two learning trials per day, as the acquisition of the MWM task with less than four trials per day is very challenging for mice (Vorhees and Williams, 2006). A previous study demonstrated improved learning performance in the MWM after environmental enrichment, which was only detected by a 2-trial a day, but not a 4-trial a day paradigm (van Praag et al., 1999). The difficulty of the task was additionally exacerbated by the presence of only very sparse visual cues.

The circular pool, 112 cm in diameter, (Stoelting) was located in a room surrounded by extra-maze (distal) cues. Water was made opaque by addition of powdered milk and water temperature was maintained at 21 ± 1°C. A cylindrical escape platform, 12 cm in diameter, made of clear plastic, was submerged 0.5 cm beneath the water surface. The maze was virtually divided by two axes (N to S and W to E) into four quadrants (NE, SE, SW, NW). The hidden platform was positioned in the middle of the SW quadrant. During six acquisition trainings mice, starting from different, pseudo-random locations around the perimeter of the tank, received two swim trials a day (max. 60 s) with an inter-trial-interval of 15 s. A probe trial (60 s) without platform was performed 24 h after the last acquisition training day. Latencies of the two daily trials are averaged.

### Data analyses

#### Statistics and numbers

All statistical information and n numbers can be found in the results part and in Supplementary Tables 2, 3. In figures, significance is indicated by asterisks (^*^p < 0.05, ^**^p < 0.01, ^***^p < 0.001). n.s. denotes non-significant results (*p* > 0.05).

#### Western blot

The intensity of the bands was analyzed using the TotalLab Quant software. Band intensities of the genes of interest were normalized to housekeeping gene GAPDH. The analyses were performed on original blots. For original blots see Supplementary Figures 2, 3.

Values for single animals and mean per treatment group are shown. Additionally the 95% confidence interval of the control group is marked as dotted lines.

#### Hearing measurements

Click- and noise-ABR measurements were analyzed by 1-way ANOVA with α = 0.05, post-test: Bonferroni-Holms. F-ABR measurements were group analyzed by 2-way ANOVA with α = 0.05, post-test: Bonferroni-Holms (GraphPad Prism). Data are shown as mean ± SD.

#### ABR analysis

For each individual ear, the peak input-output function (*peak I/O*) of the noise-ABR measurements was analyzed as previously described (Chumak et al., 2016).

Two peak classes were selected: (1) early peaks (at 1.2–1.8 ms, wave I) interpreted as the sum of the first stimulus-related action potential within the auditory nerve, and (2) delayed peaks (at 4.1–4.9 ms, wave IV), the response from the auditory midbrain. Data were analyzed by 2-way ANOVA with α = 0.05 (GraphPad Prism).

Additionally a waveform correlation analysis was performed as described in (Rüttiger et al., 2013; Singer et al., 2013). Data were analyzed by 1-way ANOVA with α = 0.05 (GraphPad Prism).

#### Electrophysiology

Data were analyzed and processed using Clampfit 10 (Molecular Devices) and Microsoft Excel. Statistics and visualization were performed with GraphPad Prism. Results between conditions were statistically compared using 1-way ANOVA and Bonferroni's Multiple Comparison Test to compare baseline vs. LTP or wash-in for both genotypes as well as LTP or wash-in between genotypes.

#### Morris water maze

Data were analyzed using Smart tracking software (Panlab) and Microsoft Excel by an experimenter unaware to the treatment of each mouse. Statistics were performed with IBM SPSS with α = 0.05. One-way repeated measures ANOVA was used to test for effect of training over time within the two treatment groups, separately. Pairwise comparisons were further performed to test for significant differences between the first training day and the following days within the two treatment groups. Two mice that did not enter the platform at least once during acquisition phase were excluded from analysis.

#### Ribbon counting

Ribbons are shown as average ribbon number per IHC (±SD). Statistical analysis was performed using 2-way ANOVA followed by a 1-tailed Student's *t*-test with α = 0.05.

#### Fluorescence analysis of brain immunohistochemistry

Pictures acquired from brain sections stained for parvalbumin (PV), were analyzed using the free software Image J. (NIH, Bethesda, MD; USA). For each section, three pictures for each single channel (YFP, CFP, PV) were saved and analyzed independently. For the 10 × magnified pictures, after conversion to an 8-bit image, background was reduced using the rolling ball algorithm (included in Image J) with standard parameters and a region of interest (ROI) of 300 × 100 μm was created and placed on the CA3 region in each single channel picture. Afterwards the average fluorescence intensity within the ROI was calculated as single pixel intensity (0–255)/no. of pixel. Data are shown as mean pixel intensity (±SEM). Data were analyzed by 1-way ANOVA with α = 0.05, post-test: Bonferroni-Holms (GraphPad Prism).

To generate representative fluorescence profile plots, a ROI of 390 × 100 μm was created and the specific Image J built-in function was used. For the 60 × magnified pictures, the same procedure was applied as for the 10 × magnified pictures within a ROI of 300 × 650 μm. To generate representative fluorescence profile plots, a ROI of 350 × 100 μm was created and the specific Image J built-in function was used.

In pictures acquired from brain sections stained for δGABA_A_-R, or α1GABA_A_-R fluorescence puncta numbers were counted using ImageJ. A 300 × 300 μm ROI was cropped, individual channels were separated and binary masks created using an appropriate thresholding algorithm for each channel. Binary particles were counted using the inbuilt Analyze Particles function.

In monochannel pictures of brain sections stained for VGLUT1 in the VCN with 100 × magnification, the mean fluorescence light intensity was measured in a frame of 85 × 60 μm (total picture) using build-in function in ImageJ.

### Data availability

The datasets generated during and/or analyzed during the current study are available from the corresponding author upon request.

## Results

### Different sound exposure conditions indicate changes in *Bdnf* exon-IV and -VI transcription that reflect sound sensitivity in the brainstem

**Figure 1:**
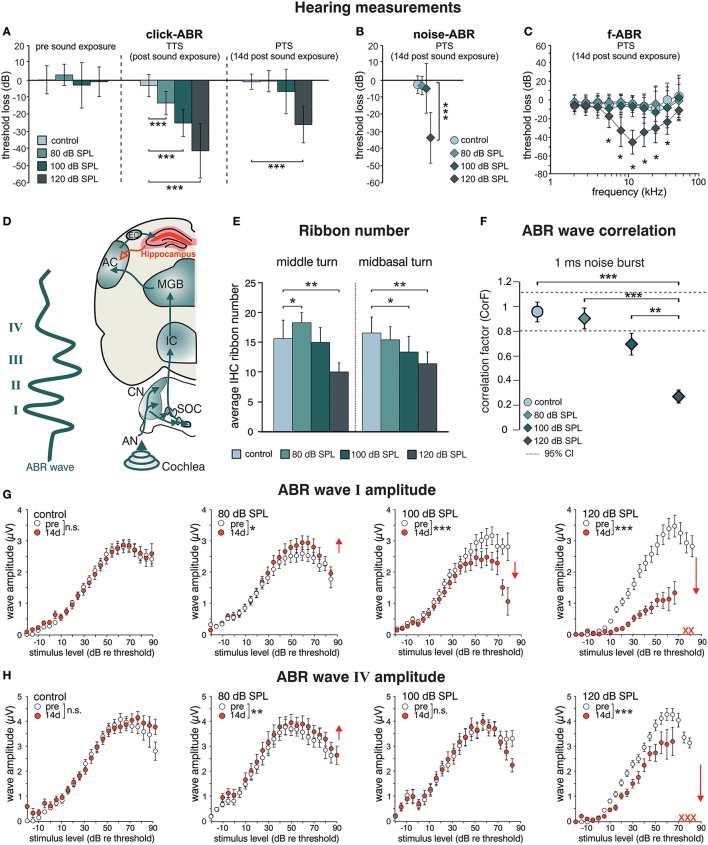
Acoustic enrichment increases hearing acuity through central adaptation while acoustic trauma leads to uncompensated hearing loss. Homozygous BLEV reporter mice were sham exposed (control) or exposed to a 10 kHz tone of 80, 100, or 120 dB SPL for 40 min. Mean ABR thresholds ± SD for click stimuli (**A**, click-ABR) before (pre sound exposure), directly after (TTS) and 14 d after sound exposure (PTS), for noise burst stimuli (**B**, noise-ABR) and for frequency-specific stimuli (**C**, f-ABR) 14 d after exposure demonstrate a significant temporary threshold shift (TTS) for all paradigms directly after exposure becoming permanent (PTS) only after 120 dB SPL sound exposure. **A–C**; con *n* = 19 animals; 80 dB SPL; *n* = 19 animals; 100 dB SPL, *n* = 16 animals; 120 dB SPL, *n* = 19 animals. **(D)** Schematic drawing of an ABR waveform in relation to the corresponding auditory nuclei in the ascending auditory pathway (green arrows) starting at the auditory nerve (AN), cochlear nucleus (CN), superior olivary complex (SOC), inferior colliculus (IC), medial geniculate body (MGB), auditory cortex (AC), and entorhinal cortex (EC) to the hippocampus as well as hippocampal projections to cortical areas (red). **(E)** IHC ribbon numbers of BLEV reporter mice for the middle and midbasal cochlear turn, representing higher frequency areas, show an increase (80 dB SPL) or decline (100 and 120 dB SPL) 14 days after sound exposure. Data represented as mean ± SD. **(F)** Analyses of ABR waveform of control animals or mice exposed to 80, 100, or 120 dB SPL 14 days after exposure. The changes in waveforms and signals were calculated as correlation factor (CorF) (Rüttiger et al., 2013). Dashed lines indicate the 95% confidence interval for the controls. ABR waveform after 120 dB SPL exposure is significantly reduced. Data represented as mean ± SEM. **(G,H)** Mean peak growth input/output function of noise burst stimulus for early peaks (**G**, ABR wave I) and late peaks (**H**, ABR wave IV) before (open circles) and 14 days after exposure (red circles) were significantly increased after 80 dB SPL exposure, significantly decreased for early peaks, but non-significantly different for late peaks after 100 dB SPL, and massively decreased after 120 dB SPL. Red crosses: early and late peaks in mice exposed to 120 dB SPL (ABR wave IV) that could not be recorded anymore. Data represented as mean ± S.E.M.

BLEV reporter mice (described in detail in Singer et al., submitted), in which translation of *Bdnf* exon-IV and -VI can be monitored by co-expression of CFP and YFP, were exposed to different sound pressure levels (SPL) inducing acoustic enrichment (80 dB SPL) and mild (100 dB SPL) or severe (120 dB SPL) acoustic trauma (Knipper et al., 2013; Singer et al., 2013). 2 weeks after exposure we observed no (80, 100 dB SPL) or moderate (120 dB SPL) loss of hearing thresholds in click-, noise-burst and frequency-specific auditory brainstem responses (ABR, Figures 1A–C). Animals exposed to 80, 100, and 120 dB SPL show a temporary threshold shift in click-ABR [TTS; Figure 1A middle panel; TTS: 1-way ANOVA: *F*_(3, 129)_ = 92.67, *p* < 0.0001, post-hoc test Bonferroni's est: con vs. 80 dB SPL *p* < 0.0001, con vs. 100 dB SPL *p* < 0.0001; con vs. 120 dB SPL *p* < 0.0001] directly after exposure, but only animals subjected to 120 dB SPL developed a permanent threshold shift [PTS; Figures 1A–C right panel; PTS: 1-way ANOVA: *F*_(3, 144)_ = 54.72, *p* < 0.0001, post-hoc test Bonferroni's test: con vs. 120 dB SPL *p* < 0.0001; noise-ABR: 1-way ANOVA: *F*_(3, 142)_ = 75.45, *p* < 0.0001, post-hoc test Bonferroni's test: con vs. 120 dB SPL *p* < 0.0001; f-ABR: 2-way ANOVA: *F*_(3, 597)_ = 79.9, *p* < 0.0001, post-hoc test Bonferroni's test: con vs. 120 dB SPL *p* < 0.05]. As observed before in rats (Singer et al., 2013), the different sound exposure paradigms manifested as long-lasting adaptations along the ascending auditory pathway (Figure 1D). These included (i) elevated (80 dB SPL, middle turn), moderately reduced (100 dB SPL, midbasal turn), or considerably reduced (120 dB SPL) numbers of CtBP2/RIBEYE-positive active release sites in the first inner hair cell (IHC) synapse [Figure 1E; 2-way ANOVA: *F*_(3, 60)_ = 11.08, *p* < 0.0001, post-hoc test 1-tailed unpaired Student's *t*-tests: middle turn: control/80 dB SPL *p* < 0.05; control/120 dB SPL *p* < 0.01; midbasal turn: control/100 dB SPL *p* < 0.05; control/120 dB SPL *p* < 0.01; *n* = 6 ears from four animals per group, 1–3 repetitions each, 8–24 IHCs per turn and group]. (ii) that the overall ABR waves' fine structure showed a loss of ABR waveform with increasing exposure levels before and 2 weeks after exposure [Figure 1F; 1-way ANOVA: *F*_(3, 131)_ = 17.51, *p* < 0.0001, post-hoc test Tukey's Multiple Comparison test: control/120 dB SPL *p* < 0.001, 80/120 dB SPL *p* < 0.001, 100/120 dB SPL *p* < 0.01; *n* = 8 animals, 15 ears (control), *n* = 9 animals, 18 ears (80 dB SPL), *n* = 5 animals, 10 ears (100 dB SPL), *n* = 9 animals, 17 ears (120 dB SPL)], which could be confirmed by detailed analyses of supra-threshold ABR waves. The amplitudes of the early supra-threshold ABR wave I [Figure 1G; ABR wave I: 2-way ANOVA; con: *F*_(1, 1031)_ = 0.003, *p* = 0.955; 80 dB SPL: *F*_(1, 890)_ = 6.02, *p* = 0.0143; 100 dB SPL: *F*_(1, 836)_ = 28.59, *p* < 0.0001; 120 dB SPL: *F*_(1, 396)_ = 185.8, *p* < 0.0001] and late ABR wave IV [Figure 1H; ABR wave IV: 2-way ANOVA, con: *F*_(1, 1034)_ = 1.296, *p* = 0.2551; 80 dB SPL *F*_(1, 951)_ = 0.89, *p* = 0.3446; 100 dB SPL: *F*_(1, 743)_ = 0.09, *p* = 0.7706; 120 dB SPL: *F*_(1, 452)_ = 82.88, *p* < 0.0001] were elevated after enriching 80 dB SPL exposure, reduced but centrally compensated after mildly traumatic 100 dB SPL exposure, and reduced for both early and late ABR waves after severely traumatic 120 dB SPL exposure (Figures 1G,H).

This indicates that sound sensitivity is persistently increased after acoustic enrichment (80 dB SPL), preserved despite reduced auditory input after mild trauma (100 dB SPL), or decreased after severe acoustic trauma (120 dB SPL). In particular the elevated IHC ribbon number and ABR wave I and IV in response to 80 dB SPL or the compensated ABR wave IV despite a reduced ABR wave I in response to 100 dB SPL exposure, can only be explained through an adaptive response that permanently alters neuronal activity in auditory pathways. Following severe auditory trauma, this adaptive response consistently failed to occur (120 dB SPL) (Figures 1G,H) as seen before in the rat model (Singer et al., 2013; Knipper et al., 2015).

In case activity-dependent *Bdnf* transcription may alter sound responsiveness by strengthening synapses via BDNF-TrkB receptor signaling, as shown in different brain regions (Kellner et al., 2014), we might expect changes in sound sensitivity to correlate with *Bdnf* transcript levels and neuronal activity. We chose vesicular glutamate transporter-1 (VGLUT1) as a marker for activity levels, as it is the predominant glutamate transporter in auditory brainstem synapses (Zhou et al., 2007).

**Figure 2:**
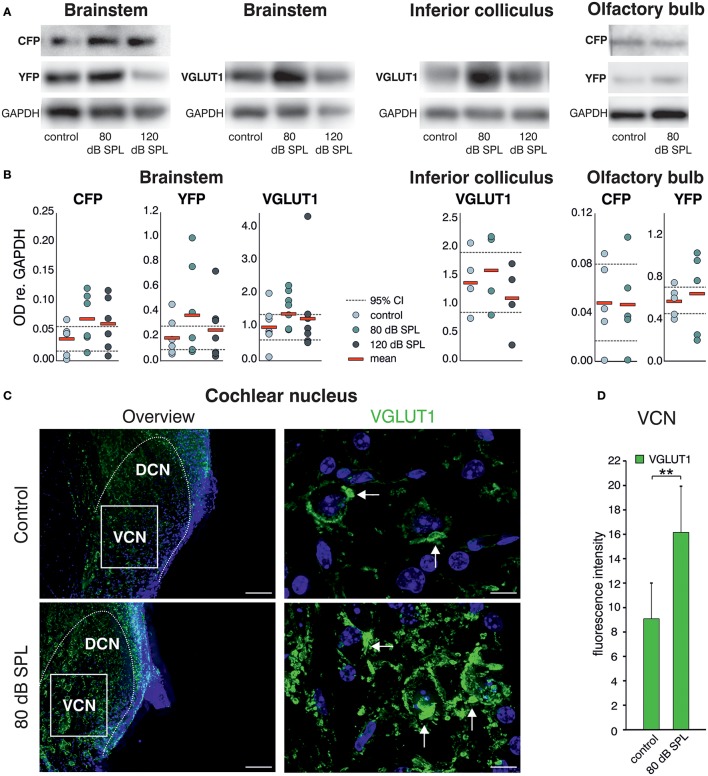
Acoustic enrichment leads to a trend for higher expression of VGLUT1 in the brainstem and inferior colliculus together with *Bdnf* exon-IV-CFP and exon-VI-YFP. **(A)** Western blot analysis of CFP and YFP in the brainstem and olfactory bulb as well as VGLUT1 in the brainstem and inferior colliculus in homozygous BLEV reporter mice 2 weeks after exposure to either sham, 80, or 120 dB SPL. The housekeeping gene GAPDH is used for normalization between conditions. For originals see Supplementary Figures 2A–C. **(B)** Densitometric analyses of Western blots (dots: averaged results per animal, bars: means, dashed lines: 95% confidence interval for the controls; *n* = 5–6 mice/group 2–10 Western blots each). **(C)** VGLUT1 immunostaining (green) in cochlear nucleus mouse slices 2 weeks after exposure to sham or 80 dB SPL. After exposure to 80 dB SPL immunoreactivity (IR) for VGLUT1 is increased in the dorsal and ventral cochlear nucleus (DCN, VCN). Nuclei stained with DAPI (blue). Arrows indicate VGLUT1 localization. Scale bars: 100 μm; 5 μm. **(D)** Quantification of VGLUT1 IR in an 85 × 60 μm frame in the VCN following sham and 80 dB SPL exposure. Data represented as mean ± SD.

Western blot (WB) analysis of *Bdnf* exon-IV-CFP and exon-VI-YFP expression levels, following immunoprecipitation with HA and cMyc tags, respectively, indicated a qualitative increase of CFP and YFP levels after acoustic enrichment (80 dB SPL), but not after acoustic trauma in the auditory brainstem (120 dB SPL; Figure 2A, left panel, see Supplementary Figure 1A for a representative selection of WBs). Consistent with elevated neuronal activity, VGLUT1 levels were qualitatively increased 2 weeks after acoustic enrichment (80 dB SPL) but not after severe acoustic trauma (120 dB SPL, Figure 2A, 2nd panel). This suggests that pathological reduction of relevant auditory input persistently prevents activity-dependent elevation of BDNF and VGLUT1. We observed a trend for mobilization of *Bdnf* exon-IV-CFP and exon-VI-YFP and VGLUT1 not only in the auditory brainstem, but also in the inferior colliculus (Figure 2A, 3rd panel, see Supplementary Figure 1B for a representative selection of WBs). However, due to the highly variable immunoprecipitation yield, quantification of WB analyses did not reach statistical significance (Figure 2B). Importantly, following enriched sound no indications for mobilization of transcript-specific BDNF in the olfactory bulb was observed, as shown for *Bdnf* exon-IV-CFP and *Bdnf* exon-VI-YFP (Figure 2A, right panel). To quantitatively verify long-lasting changes of neuronal activity in the target cells of the auditory nerve, we additionally analyzed VGLUT1 immunoreactivity (IR) in bushy cells of the cochlear nucleus (CN) within the auditory brainstem 2 weeks after acoustic enrichment [80 dB SPL; Figures 2C,D; two-tailed Student's *t*-test: *t* = 3.63 *df* = 10 *p* = 0.0046; *n* = 6 mice/group 2–3 repetitions]. A significantly elevated punctate VGLUT1 IR at the level of CN bushy cells, exemplarily shown in Figure 2C and quantified in Figure 2D, was observed in comparison to sham (control) exposure. This suggested that 2 weeks following 80 dB SPL exposure, auditory nerve synapses may exhibit larger numbers of active release sites or greater spike fidelity, considered as a functional correlate of elevated VGLUT1 following sound exposure (Ngodup et al., 2015).

In summary, we observed increased expression of VGLUT1 together with a trend for upregulation of *Bdnf* exon-IV-CFP and exon-VI-YFP in the auditory brainstem in animals with elevated early (auditory nerve) or late (brainstem) supra-threshold auditory responses, but not in animals with critically reduced supra-threshold auditory nerve responses that failed to be centrally restored. This is consistent with the idea that a relevant auditory input drives *Bdnf* transcription and subsequently elevates levels of BDNF in the brainstem, which might alter VGLUT1 levels, as previously suggested for hippocampal neurons. This could strengthen synapses which depend on the activity of BDNF (Kellner et al., 2014). Comparable changes in *Bdnf* transcription were not detected in other sensory regions like the olfactory bulb, indicating specificity of activity-dependent BDNF transcription following auditory inputs.

### Enriching sound exposure indicates changes in hippocampal *Bdnf* transcription correlating with increased synaptic plasticity and improved memory acquisition

**Figure 3:**
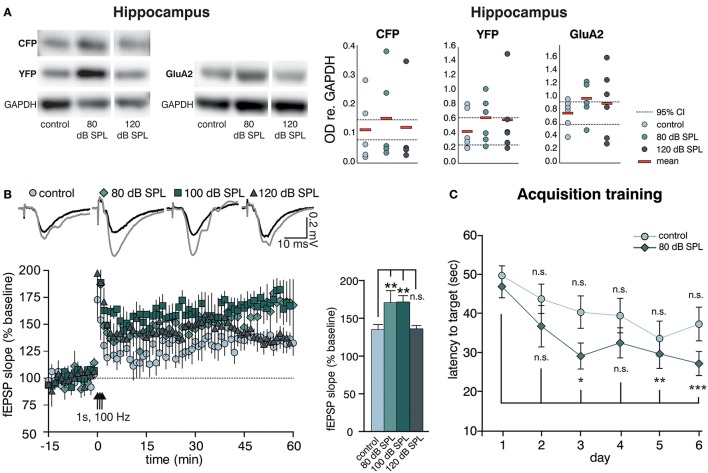
Acoustic enrichment enhances hippocampal BDNF expression and LTP as well as memory acquisition in the Morris water maze (MWM). **(A)** Left panel: Western blot analysis of *Bdnf* exon-IV-CFP and exon-VI-YFP (left blot) as well as GluA2 (right blot) in the hippocampus of BLEV reporter mice exposed to sham, 80, or 120 dB SPL. Right panel: Densitometric analyses of Western blots (dots: averaged results per animal, bars: means, dashed lines: 95% confidence interval for the controls; *n* = 5 mice/group 2–10 Western blots each). For originals see Supplementary Figure 2D. **(B)** Left Panel: Averaged time courses of fEPSP slopes in acute brain slices from BLEV reporter mice 2 weeks after exposure to sham, 80, 100, or 120 dB SPL. Representative traces before (black) and after (gray) induction of LTP are shown on top. Animals from all conditions readily show significant SC LTP. Right panel: Significantly increased LTP was observed for 80 (171 ± 16% of baseline) and 100 (172 ± 8% of baseline) but not 120 dB SPL (136 ± 4% of baseline) compared to controls (135 ± 7% of baseline). Data are represented as mean ± SEM. **(C)** Latency to target during acquisition learning. After 3 days mice exposed to 80 dB SPL need less time to find the platform in comparison to the first experimental day than mice in the control group. Data are expressed as mean ± SEM.

We previously observed altered levels of Arc (activity-regulated cytoskeletal protein) in the hippocampal CA1 region after acoustic enrichment, as well as after mild and severe acoustic trauma in rats (Singer et al., 2013). Arc plays a key role in determining synaptic strength through facilitation of AMPA receptor (AMPAR) endocytosis in response to BDNF signaling (Bramham et al., 2008; Wall and Corrêa, 2017). Similarly, as previously reported for Arc (Singer et al., 2013), we observed a trend for an increase in the hippocampal expression of another excitability marker, the GluA2 subunit of the AMPAR (Tanaka et al., 2000; Singer et al., 2013) 2 weeks after acoustic enrichment, but not after severe acoustic trauma in BLEV reporter mice (Singer et al., 2013). This elevation of hippocampal excitability was paralleled by a trend for higher levels of *Bdnf* exon-IV-CFP and exon-VI-YFP (Figure 3A, first panel, Supplementary Figure 1C for a representative selection of WBs) which was likewise only observed after acoustic enrichment, but not after severe acoustic trauma. These findings are in line with different sound exposure conditions driving adaptations of *Bdnf* transcript levels and glutamatergic neuronal activity not only in the brainstem but also in the hippocampus.

Modulation of BDNF-dependent Arc expression was previously associated with increased hippocampal synaptic plasticity (Kuipers et al., 2016), particularly with formation of long-term potentiation (LTP) (Messaoudi et al., 2007). Therefore, we went on to test if the altered hippocampal BDNF expression levels and neuronal excitability changes observed after acoustic enrichment or acoustic trauma also influenced hippocampal synaptic plasticity in the BLEV reporter mouse. To this end, we recorded field excitatory postsynaptic potentials (fEPSP) in the CA3 to CA1 Schaffer collateral (SC) synapses in the stratum radiatum (SR) from acute forebrain slices of BLEV reporter mice 2 weeks after sham (control) treatment, acoustic enrichment, as well as mild and severe acoustic trauma [Figure 3B, Supplementary Figure 4; 1-way ANOVA: *F*_(3, 19)_ = 4.99, *p* = 0.01, post-hoc test Bonferroni's test baseline/tetanized (b/t) *n* = 4 animals/group con: 7 slices, 80 dB SPL: 7 slices, 100 dB SPL: 6 slices, 120 dB SPL: 5 slices, control *p* < 0.01, 80 dB SPL *p* < 0.001, 100 dB SPL *p* < 0.001, 120 dB SPL *p* < 0.01; tetanized/tetanized (t/t) con vs. 80 dB SPL *p* < 0.01 con vs. 100 dB SPL *p* < 0.01 con vs. 120 dB SPL n.s.]. None of the sound exposure paradigms led to changes in basal synaptic transmission, as all four conditions displayed similar fEPSP amplitudes in response to a range of input strengths (Supplementary Figure 4A). Additionally, similar levels of paired-pulse facilitation in all four conditions indicated no changes in presynaptic function (Supplementary Figure 4B). We were able to observe significant LTP in acute brain slices from mice under all four conditions in response to tetanic stimulation (1 s, 100 Hz). This potentiation, however, was significantly stronger in animals exposed to acoustic enrichment or mild acoustic trauma as compared to animals exposed to sham or severe acoustic trauma (Figure 3B). This finding suggests that persistently improved (after acoustic enrichment) or restored (after mild acoustic trauma) sound responses leads to altered *Bdnf* exon-IV-CFP and exon-VI-YFP expression as well as synaptic excitability and plasticity in the brainstem and hippocampus. Reduced sound responses after severe acoustic trauma, on the other hand, forestall activity-dependent BDNF expression, improved excitability, and synaptic plasticity.

We wanted to know next if elevated hippocampal BDNF expression not only correlates with increased GluA2 levels and SC LTP, but also with performance in hippocampus-dependent learning. To test this, starting 10 days after sound exposure, we subjected mice exposed to sham treatment (control) or acoustic enrichment (80 dB SPL) to a Morris water maze (MWM) task, which is a hippocampus-dependent learning paradigm. In order to identify increased learning performance (van Praag et al., 1999), we opted for a very challenging paradigm that included only two trials per day (for details, see Methods).

While control mice improved slightly but not significantly over time performing the task, acoustically enriched mice significantly improved their performance upon training. This was evidenced by reduced latencies in finding the platform as compared to the first day [Figure 3C; repeated measure ANOVA: con: *F* = 2.56 DF = 5 *p* = 0.033; post-hoc test Bonferroni's test: day 1 vs. 2 n.s., day 1 vs. 3 n.s., day 1 vs. 4 n.s., day 1 vs. 5 n.s., day 1 vs. 6 n.s.; 80 dB SPL: *F* = 5.85 *DF* = 5 *p* < 0.001; Bonferroni's test: day 1 vs. 2 n.s., day 1 vs. 3 *p* = 0.013, day 1 vs. 4 *p* = 0.054, day 1 vs. 5 *p* = 0.006, day 1 vs. 6 *p* < 0.001; con *n* = 18 animals 80 dB SPL *n* = 17]. We could not detect any correlation between MWM performance and gender or age.

So far, the presented data indicate that acoustic enrichment leads to elevated expression of *Bdnf* exon-IV-CFP and exon-VI-YFP together with excitatory markers in the auditory brainstem and the hippocampus, paralleled by increased hippocampal LTP and improved performance in a hippocampus-dependent learning paradigm. Neither sound-induced improval of auditory performance nor increased hippocampal LTP are observed in animals exposed to severe acoustic trauma, in which activity-dependent *Bdnf* transcript levels are not elevated in response to auditory sound exposure.

### Acoustic enrichment and mild, but not severe trauma, synchronously alter hippocampal *Bdnf* exon-IV and exon-VI transcription in neuronal and vascular cells in parallel to parvalbumin expression

**Figure 4:**
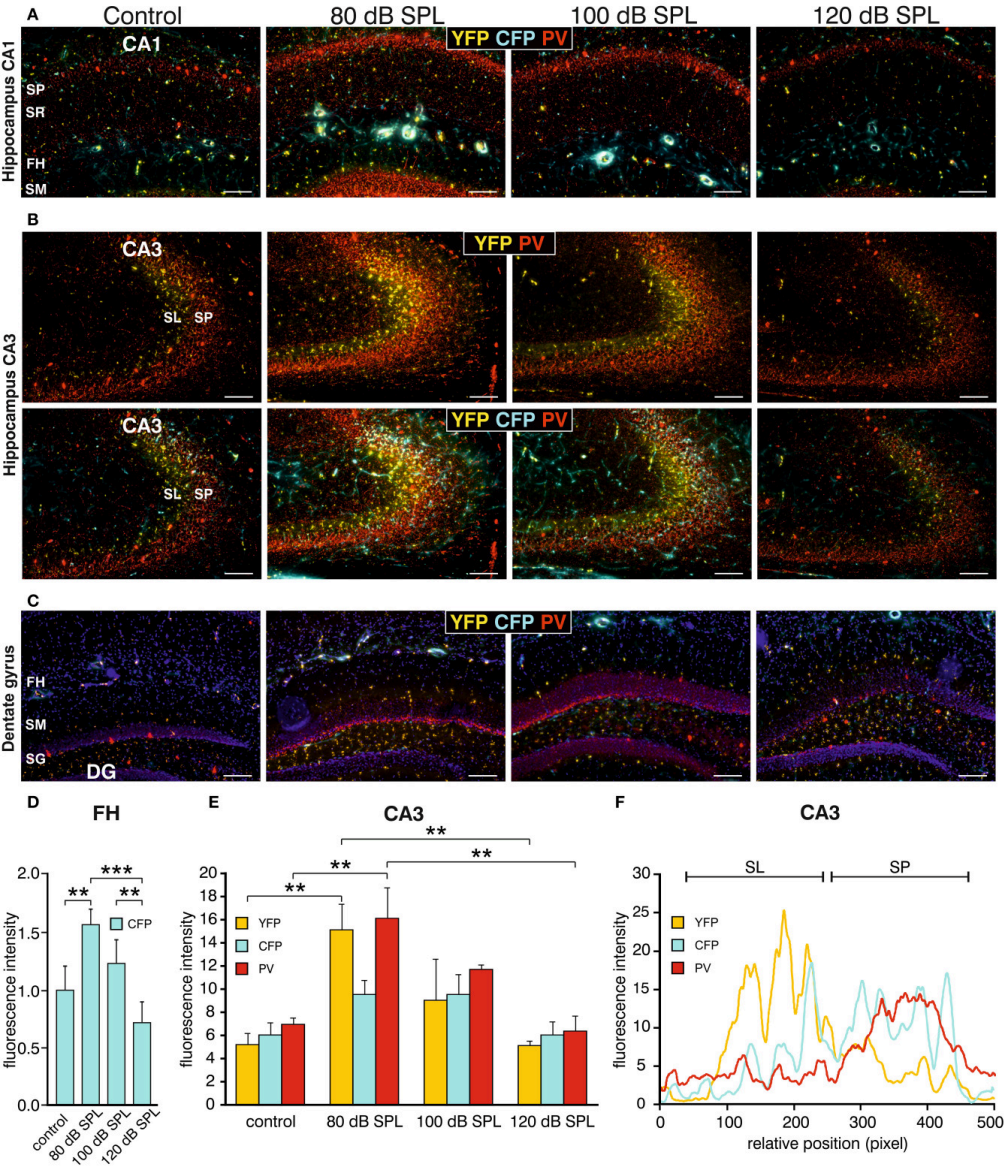
Dynamic changes of *Bdnf* exon-IV and -VI transcripts and parvalbumin upon sound exposure in hippocampal circuits (Supplementary Video 1). **(A–C)** CFP/YFP fluorescence and parvalbumin immunostaining (PV, red) in dorsal hippocampal mouse slices 2 weeks after exposure to sham, 80, 100, or 120 dB SPL. Note a prominent dynamic change of PV (red) expression in the stratum pyramidale (SP) of the CA1 region **(A)**, in the stratum lucidum (SL) of the CA3 region (*n* = 6 animals/group) **(B)**, and stratum granulosum (SG) of the dentate gyrus (DG) **(C)** concomitant to distinct changes in YFP and CFP. SM, stratum moleculare; SR, stratum radiatum; FH, fissura hippocampalis. Scale bars, 100 μm. **(D)** Quantification of *Bdnf* exon-IV-CFP in capillary outlines at the level of the FH revealed a peak of fluorescence in 80 dB SPL exposed animals that declined below control levels in 120 dB SPL exposed animals. Data represented as mean ± SD. **(E)** Quantification of YFP, CFP, and PV intensities in the CA3 region in an area of 300 × 100 μm following different sound exposure paradigms. Data represented as mean ± SEM. **(F)** Intensity profile of CFP, YFP, and PV along a line through the CA3 of a control mouse, perpendicular to the SP (length: approximately 390 μm). Representative for four experiments.

We subsequently took advantage of the unique utility of the BLEV reporter mouse to identify the cell types where activity-dependent *Bdnf* transcription occurs in response to sound exposure. As inhibitory transmission is a major regulator of hippocampal synaptic plasticity (Matt et al., 2011) and BDNF is known to influence the function of parvalbumin (PV)-positive inhibitory interneurons (Marty, 2000; Yamada and Nabeshima, 2003; Messaoudi et al., 2007; Hong et al., 2008; Minichiello, 2009; Waterhouse et al., 2012; Park and Poo, 2013), we asked if changes in activity-dependent *Bdnf* transcript usage might be observed in neurons or cells known to target PV-positive interneurons. For example, mossy fibers express BDNF and are suggested to target PV-positive interneurons (Danzer et al., 2008; Dieni et al., 2012). We first examined *Bdnf* exon-IV-CFP, exon-VI-YFP, and PV expression in deconvoluted high-resolution fluorescence stacks of low-magnification (Figure 4) in the CA1 region (Figure 4A; Supplementary Video 1), the CA3 region (Figure 4B; Supplementary Video 1), and the dentate gyrus (Figure 4C) of the hippocampus. Compared to sham exposed animals (control), we observed a general upregulation of CFP, YFP, and PV (red) in all regions of the hippocampus after acoustic enrichment and mild acoustic trauma, the two conditions associated with increased hippocampal synaptic plasticity (Figure 3B, Supplementary Figure 4). In control animals, CFP fluorescence is mostly seen in capillary vessels of the highly vascularized fissura hippocampalis (FH) (Figure 4A). These CFP levels in the FH were significantly higher after acoustic enrichment and mild acoustic trauma but not after severe acoustic trauma [Figure 4D; 1-way ANOVA: *F*_(3, 19)_ = 10.5, *p* < 0.0003, post-hoc test Bonferroni's test: con vs. 80 dB SPL, *p* = 0.0013, con vs. 100 dB SPL n.s. con vs. 120 dB SPL n.s., 80 dB SPL vs. 120 dB SPL *p* < 0.0001, 100 dB SPL vs. 120 dB SPL *p* = 0.0072; con: *n* = 6 animals, 1–4 repetitions; 80 dB SPL *n* = 6 animals, 1–6 repetitions; 100 dB SPL *n* = 5 animals, 2–5 repetitions; 120 dB SPL *n* = 6 animals, 1–3 repetitions]. In the CA1 region of animals exposed to acoustic enrichment or mild acoustic trauma, we observed increased PV labeling in the CA1 pyramidal layer (Figure 4A, SP). In the CA3, we found strongly amplified signals of both PV and *Bdnf* exon-VI-YFP in the area where mossy fibers are predicted to target CA3 dendrites (Figures 4B,E). PV labeling was also increased in the supra-pyramidal blade of the DG (Figure 4C). This generalized pattern of up-regulation was not observed in animals exposed to 120 dB SPL (Figures 4A–E). Particularly within the stratum lucidum (SL) of the CA3 region (Figure 4B), the increase in *Bdnf* exon-VI-YFP expression, together with PV, was either robust (80 dB SPL), moderate (100 dB SPL), or absent [120 dB SPL, Figure 4E; YFP: 1-way ANOVA: *F*_(3, 11)_ = 6.96, *p* = 0.0068, post-hoc test Bonferroni's test con vs. 80 dB SPL *p* < 0.01, 80 dB SPL vs. 120 dB SPL *p* < 0.01; CFP: 1-way ANOVA *F*_(3, 11)_ = 2.37, *p* = 0.13; PV: 1-way ANOVA *F*_(3, 11)_ = 10.07, *p* = 0.0017, post-hoc test Bonferroni's test con vs. 80 dB SPL *p* < 0.01, 80 dB SPL vs. 120 dB SPL *p* < 0.01; con: *n* = 4 animals; 80 dB SPL *n* = 3 animals; 100 dB SPL *n* = 3 animals; 120 dB SPL *n* = 5 animals, 3 repetitions each]. Fluorescence intensity profiles taken through the SL and SP of CA3 of a control mouse revealed that YFP fluorescence was predominantly found in the SL, while CFP and PV fluorescence was restricted to the SP (representative profile plot in Figure 4F).

Consistent with our previous observations in terms of ABR wave amplitudes, the expression of excitatory markers in the auditory brainstem, the inferior colliculus, and the hippocampus, as well as hippocampal LTP and learning, we thus also confirmed a correlation between PV expression patterns and changes of *Bdnf* exon-IV-CFP and exon-VI-YFP levels in the tri-synaptic pathway.

**Figure 5:**
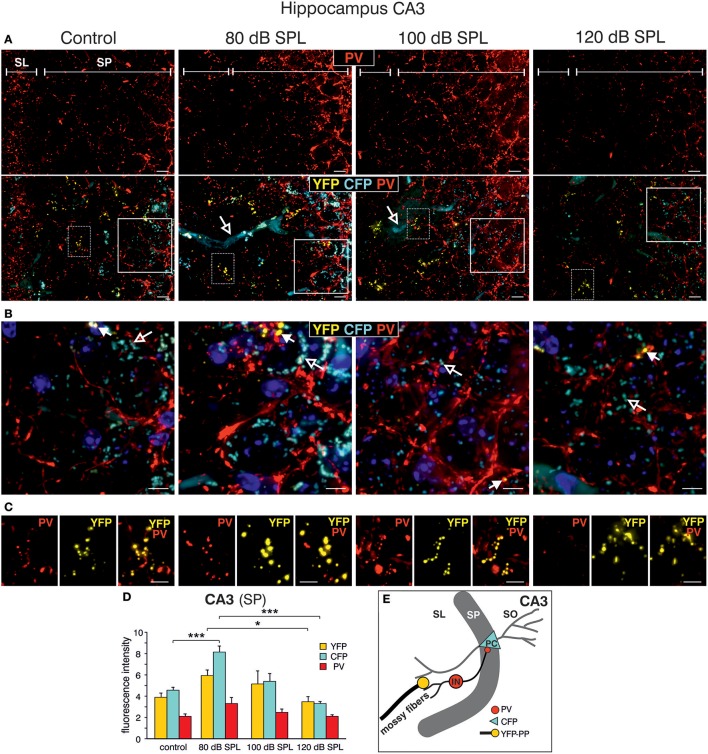
Dynamic changes of *Bdnf* exon-IV and -VI transcripts and parvalbumin expression in the CA3 region following sound exposure. **(A,B)** Hippocampal CA3 region with magnification of the stratum lucidum (SL) and stratum pyramidale (SP). **(A)** Immunostaining of parvalbumin (PV, red). Note the difference in PV expression in the SP in dependence of the applied sound exposure. Scale bars: 10 μm. Lower panel: Triple staining of the same CA3 region. Open arrows indicate *Bdnf* exon-IV-CFP in blood vessels. Scale bars: 10 μm. **(B)** Magnified view from solid frame in **(A)**. CFP (open arrows) and PV (red) expression at the level of the SP. Closed arrows indicate *Bdnf* exon-VI-YFP. Nuclei stained with DAPI (blues). Scale bars: 5 μm. **(C)** Magnified view from dashed frame in **(A)**. *Bdnf* exon-VI-positive-YFP expression in mossy fiber terminals contacts overlaps with PV-positive (red) interneurons in the SL region of the CA3 and increases (80 dB SPL) or declines (120 dB SPL) with sound. Scale bars: 5 μm. **(A–C)** *n* = 6 animals/group. **(D)** Quantification of CFP, YFP, and PV fluorescence averaged over the SL and SP region of the CA3 region. *Bdnf* exon-IV-CFP fluorescence intensity peaks in animals subjected to 80 dB SPL. *Bdnf* exon-VI-YFP and *Bdnf* exon-IV-CFP after 80 dB SPL sound exposure were also significantly elevated when compared to 120 dB SPL-exposed animals. Data represented as mean ± SD. **(E)** Model depicting assumed locations of altered CFP, YFP, and PV expression after 80 dB SPL sound exposure. Mossy fiber terminals (yellow) terminate on dendrites of excitatory CA3 pyramidal cells (PC) and PV-positive inhibitory interneuron (IN, red). CFP is found in the perisomatic areas of CA3 PCs (blue). SO, stratum oriens.

This finding led us to perform a more detailed analysis of CFP, YFP, and PV expression in the hippocampal CA3 region at a high-magnification. CFP fluorescence was mainly restricted to blood vessels in the SL (Figure 5A, lower panels, open arrows) and to perisomatic regions in the SP (Figure 5B, open arrows). YFP fluorescence was predominantly found in the SL of the CA3 region (Figure 5A, lower panels; Figure 5B, closed arrows). In all these areas, fluorescence intensity was increased after acoustic enrichment and mild acoustic trauma, but not after severe acoustic trauma. Many of the YFP-positive puncta in the SL overlapped with PV IR (Figure 5C), indicating that YFP positive terminals not only contact dendrites of pyramidal cells (PCs) but also those of PV-positive interneurons (Figure 5E). This suggests that *Bdnf* exon-IV translation in mossy fiber terminals (Danzer et al., 2008; Zheng et al., 2011; Dieni et al., 2012) in response to enriched or mild traumatic sound is linked to elevated levels of PV in the perisomatic area of CA3 pyramidal neurons (Figure 5B, red). Similarly as observed above, PV IR was strongly increased after acoustic enrichment and mild acoustic trauma, but not after severe acoustic trauma (Figure 5B). Quantification of fluorescence intensity in the SP revealed an increase of CFP fluorescence after acoustic enrichment in comparison to controls and a reduction between enriching and severe acoustic trauma. For YFP intensity, a significant reduction between enriching and severe acoustic trauma was observed [Figure 5D; CFP: 1-way ANOVA: *F*_(3, 25)_ = 22.44, *p* < 0.0001, post-hoc test Bonferroni's test con vs. 80 dB SPL *p* < 0.001 80 dB SPL vs. 120 dB SPL *p* < 0.0001; YFP: 1-way ANOVA: *F*_(3, 25)_ = 3.32, *p* = 0.036 post-hoc test Bonferroni's test 80 dB SPL vs. 120 dB SPL *p* < 0.05 *n* = 6 animals/group; 4–6 repetition each]. These findings suggest that BLEV mice can be used to identify mossy fiber terminals and CA3 projection neurons that respond to defined behaviorally relevant sensory stimuli.

### Acoustic enrichment but not severe acoustic trauma decreases dendritic inhibition of CA1 pyramidal cells

**Figure 6:**
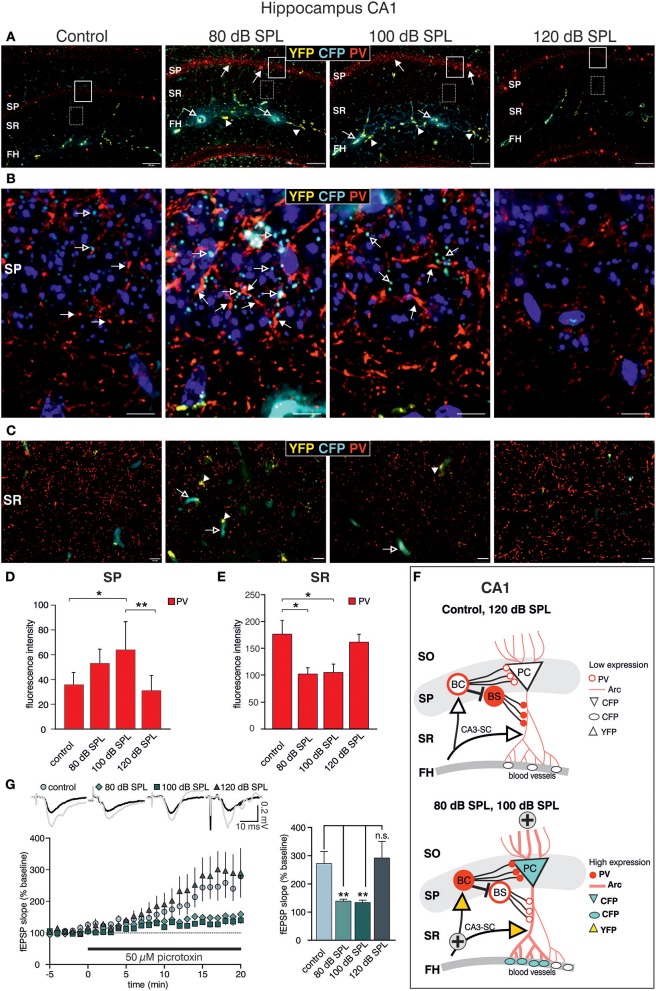
Dynamic changes of *Bdnf* exon-IV and -VI transcripts and parvalbumin after sound exposure in the hippocampal CA1 region. **(A)** Low-magnification of parvalbumin (PV) IR in BLEV reporter mice 2 weeks after exposure to control, 80, 100, or 120 dB SPL. Note the up-regulation of PV IR (arrows) in the SP region parallel to *Bdnf* exon-IV-CFP (open arrows) in the vascularized FH region. Scale bars. 100 μm. **(B)** High-power magnification of PV IR in regions framed exemplarily in **(A)** unravels an increase in PV-immunopositive perisomatic contacts (closed arrows) concomitant to *Bdnf* exon-IV-CFP expression (open arrows) in a somatic localization at the SP level. Scale bars: 20 μm. **(C)** PV IR in the SR, see frames in exemplarily region in **(A)**, indicating reduced PV-immunopositive puncta in BLEV reporter mice 2 weeks after exposure to 80 and 100 dB SPL in comparison to control or 120 dB SPL. Scale bars: 100 μm. **(A–C)** *n* = 6 animals/group. **(D)** Quantification of PV-immunopositive puncta at the SP level are elevated for 80 and 100 dB SPL exposed animals. Data are represented as mean ± SD. **(E)** The quantification of PV-immunopositive puncta within the SR revealed a significant decline of PV-immunopositive dots for 80 and 100 dB SPL- and unchanged levels in 120 dB SPL-exposed animals. Data are represented as mean ± SEM. **(F)** Abstract figure of the CA1 region indicating the expression pattern of Arc, PV, CFP, and YFP. ⊕ Indicates increased activity. FH, Fissura hippocampalis; SO, Stratum oriens; SP, Stratum pyramidale; SR, Stratum radiatum; PC, pyramidal cell; BC, basket cell; BS, bistratified cell, SC, Schaffer collaterals. **(G)** Averaged time courses of picrotoxin wash-in experiments. Representative traces before (black) and after (gray) wash-in of 50 μM picrotoxin are shown on top. Wash-in of picrotoxin leads to an increase in fEPSP amplitude. This disinhibition is significantly stronger in controls (272 ± 43% of baseline) and 120 dB SPL (292 ± 59% of baseline) compared to 80 dB SPL (139 ± 7% of baseline) and 100 dB SPL (134 ± 8% of baseline). Data are represented as mean ± SEM.

We next examined high-magnification images of the CA1 area. Following acoustic enrichment and mild trauma, CFP fluorescence increased in the FH (Figure 6A, open arrows), in perisomatic areas of the SP (Figure 6B, open arrows), and in blood vessels of the SR (Figure 6C, open arrows). YFP fluorescence on the other hand was increased in the FH and the SR after acoustic enrichment and mild acoustic trauma close to capillaries (**Figures 6A,C**, arrowheads) (Marosi and Mattson, 2014; Miyamoto et al., 2014). Similar to CA3, perisomatic PV IR (red) in the SP of CA1 was significantly increased after acoustic enrichment and mild acoustic trauma [Figure 6A; Figure 6B, closed arrows; Figure 6D, quantification; 1-way ANOVA: *F*_(3, 19)_ = 5.96, *p* = 0.0049, post-hoc test Bonferroni's test: con vs. 80 dB SPL n.s.; con vs. 100 dB SPL, *p* = 0.0301; con vs. 120 dB SPL n.s; 100 vs. 120 dB SPL *p* = 0.01; con *n* = 6 animals, 80 dB SPL *n* = 6 animals, 100 dB SPL *n* = 5 animals, 120 dB SPL *n* = 6 animals; 4–6 repetitions]. In the SR, however, we found a significant decrease of PV IR after acoustic enrichment and mild acoustic trauma [Figure 6C, red IR; Figure 6E, quantification; 1-way ANOVA: *F*_(3, 19)_ = 4.61, *p* = 0.0138, post-hoc test Bonferroni's test: con vs. 80 dB SPL *p* < 0.05, con vs. 100 dB SPL *p* < 0.05, con *n* = 6 animals 80 dB SPL *n* = 5 animals, 100 dB SPL *n* = 6 animals, 120 dB SPL, *n* = 6 animals; 2 repetitions each]. After severe acoustic trauma, we once more could not detect any differences in CFP and YFP fluorescence as well as PV IR in comparison to controls (Figures 6A–E).

These observations point to an increased perisomatic inhibitory connection in the SP derived from PV-positive interneurons and a simultaneous decrease of such connections in the SR. PV-positive interneurons target CA1 PC through perisomatic δ subunit containing GABA_A_-receptors and its dendrites through α1 subunit containing GABA_A_-receptors (Klausberger et al., 2003; Glykys et al., 2008) (Figure 6F). Indeed, we found that puncta of PV IR in the CA1 SP often co-localized with IR for the δ subunit containing GABA_A_-receptor (Supplementary Figures 5A,B) and that α1 subunit containing GABA_A_-receptor IR was localized in the CA1 SR (Supplementary Figure 5D). After acoustic enrichment and mild acoustic trauma, however, no significant changes of δGABA_A_ receptor-immunopositive dots (Supplementary Figures 5C,E) or of α1GABA_A_-receptor IR in the CA1 SR (Supplementary Figures 5D,F) were detected.

As α1 subunit containing GABA_A_-receptors in the CA1 SR are suggested to regulate excitability and action potential thresholds of PC dendrites (Willadt et al., 2013) a functional test of dendritic inhibition in the CA1 SR was performed [**Figure 6G;** 1-way ANOVA: *F*_(3, 19)_ = 5.24, *p* = 0.005, post-hoc test Bonferroni's test: con *p* < 0.001, 80 dB SPL n.s., 100 dB SPL n.s., 120 dB SPL *p* < 0.001; con vs. 120 dB SPL *p* < 0.01, con vs. 80 dB SPL *p* < 0.01, con vs. 100 dB SPL n.s.; baseline/wash-in *n* = 4 animals/group; 9 slices/group]. We observed SC fEPSP during wash-in of the GABA_A_ receptor antagonist picrotoxin. The ensuing disinhibition was significantly decreased in mice exposed to acoustic enrichment and mild acoustic trauma (Figure 6G), which is consistent with the downregulation of PV-positive terminals (Figures 6C,E) and a slight decrease in α1 subunit containing GABA_A_-receptor expression in the CA1 SR (Supplementary Figure 5D, green), compared to animals exposed to sham treatment or severe acoustic trauma. Additionally, this decrease in dendritic inhibition may serve as an explanation for increased LTP and learning capability observed after acoustic enrichment and mild acoustic trauma but not after severe acoustic trauma (Figures 3B,C, 6F).

**Figure 7:**
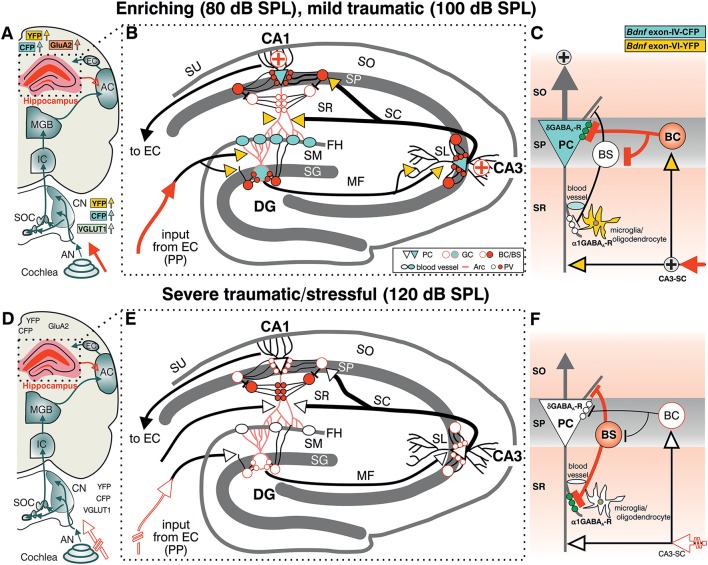
Summary of the location pattern of CFP (blue), YFP (yellow), neuronal (PV) and activity markers (Arc) in the hippocampus. Depicted is the situation in response to enriching or mild traumatic events (80/100 dB SPL) **(A–C)** or to severe traumatic/stressful events (120 dB SPL) **(D–F)**. **(A,D)** Schematic drawing of the auditory pathway including hippocampal connections (red open arrow). **(B,E)** Schematic drawing of hippocampus with perforant path (PP). **(C,D)** Schematic drawing of dendritic (BS) and somatic (BC) inhibitory inputs onto a pyramidal cell (PC). CFP, YFP, VGLUT1 (**A**, light green), GluA2 (**A**, light orange), Arc (**B** red dendrites), PV (**B** red filled circles), and the δ subunit containing GABA_A_ receptors in PCs (green dots in **C**) were shown to be mobilized after exposure to 80 or 100 dB SPL **(A–C)** but not to 120 dB SPL **(D–F). (C,F)** The cooperative transcript-specific BDNF release may drive a long-lasting reduction of dendritic (**C**, α1 subunit containing GABA_A_ receptors, white small dots) and increase of perisomatic inhibition (**C**, δ subunit containing GABA_A_ receptors, green small dots) of CA1 PC by PV expressing inhibitory interneurons (BS, BC) and in parallel possibly improve the recruitment of vascularization (**B,E**, blood vessels). This central facilitating or adaptive responsiveness (**A–C**, solid red arrow indicating increased input activity) is severely hampered when a critical damage of peripheral auditory input occurs (**D–F** discontinuous red lined arrow). ⊕ symbol indicates increased output activity in 80 or 100 dB SPL exposed animals **(C)**. Green dots in **(C)** represent δ subunit containing GABA_A_ receptors in SP, green dots in **(F)** represent α1 subunit containing GABA_A_ receptors in SR. AC, auditory cortex; AN, Auditory nerve; BC, basket cell; BS, bistratified cell; CA3-SC, excitatory input from CA3 Schaffer collaterals; CN, cochlear nucleus; DG, dentate gyrus; EC, entorhinal cortex; FH, fissura hippocampalis; GC, granule cell; IC, inferior colliculus; MF, mossy fibers; SC, Schaffer collaterale; SG, stratum granulare; SL, stratum lucidum; SM, stratum moleculare; SO, stratum oriens; SOC, superior olivary complex; SP, stratum pyramidale; SR, stratum radiatum; SU, subiculum.

Taken together, the present findings suggest that exposure to acoustic enrichment (80 dB SPL) and mild acoustic trauma (100 dB SPL), conditions that increase sound sensitivity, hippocampal LTP, and learning appears to correlate with an elevation of *Bdnf* exon-IV-CFP and *Bdnf* exon-VI-YFP in the brainstem and hippocampus. In the hippocampus, sound-driven increase of *Bdnf* exon-VI-YFP levels reaches mossy fiber terminals and hippocampal capillaries where an elevation of *Bdnf* exon-IV-CFP is observed. In addition, *Bdnf* exon-IV-CFP in the pyramidal layer is activated in response to sound and within CA1 PC that receive more perisomatic, but a reduced number of dendritic PV-positive contacts (Figures 7A–C). In contrast, a reduction of auditory input after severe acoustic trauma (Figures 7D–F, discontinuous red lined arrow) showed no apparent mobilization of *Bdnf* exon-VI-YFP and *Bdnf* exon-IV-CFP transcripts, neither in the brainstem, nor in neuronal or capillary hippocampal cells. Under these conditions, we could not observe compensatory adaptation of sound sensitivity, nor increased hippocampal LTP (Figures 7D–F). These findings suggest that a crucial level of auditory input drives activity-dependent transcription of BDNF restricted to the ascending auditory pathway and the hippocampus to establish persistent adaptation of the auditory sensory system. Using the BLEV reporter mouse, we will now be able to identify the neuronal and non-neuronal cell populations within the brainstem and hippocampus that guide this behaviorally relevant adaptation process.

## Discussion

We here provide evidence that BLEV mice allow specific identification of neurons and capillaries that respond to a critical behaviorally relevant sensory input by elevation of exon-IV and -VI derived *Bdnf* expression. Our data indicate that a sound-induced and memory-dependent improvement or restoration of sound sensitivity may take place in the auditory pathway and hippocampus. In contrast, an impairment of the critical auditory input after severe auditory trauma apparently leads to a failure to elevate *Bdnf* transcripts and to adapt to sound sensitivity. Therapeutic concepts for maladaptive diseases therefore need to reconsider BDNF replacement strategies in view of the present findings.

### Acoustic enrichment persistently increases responsiveness of the auditory system coinciding with increased excitability and translation of *Bdnf* exon-IV and exon-VI

In the present study, different levels of acoustic exposure induced persisting changes in sound-sensitivity that apparently correlated with changes of *Bdnf* exon-IV-CFP and VI-YFP levels in the brainstem and hippocampus. Considering the driving force for these transcript-specific BDNF changes we have to take into account that these external sound exposure conditions, here presented by (novel) experience of enriching, mild traumatic, or severe traumatic sound for 40 min, will not only activate the auditory path but will also drive an immediate glucocorticoid (GC)-mediated stress response associated to arousal or fear (De Kloet et al., 2005; de Kloet, 2014; Hermans et al., 2014; Myers et al., 2014). In line with this assumption, the cortisol level is elevated 30 min after sound exposure onwards (Campeau and Watson, 1997). Likewise, elevated cortisol levels influence IHC synapses contacting postsynaptic auditory fibers as well as sound responses of afferent auditory fibers (Singer et al., 2013). Additionally, an acute behavioral stress paradigm and glucocorticoid receptor antagonists influence sound-induced suprathreshold auditory nerve responses, indicating that circulating cortisol reaches the cochlea via the bloodstream (Singer et al., 2013, 2018). Moreover, the same behavioral stress paradigm triggered sound-enrichment driven elevated levels of the LTP-associated activity-regulated cytoskeletal protein (Arc) in the hippocampus (Singer et al., 2013). On the other hand, stress paradigms that lead to high corticosterone blood levels, elevate the risk to reduce central auditory responses after acoustic trauma and reduced Arc levels (Singer et al., 2013). Overall, these previous studies support the hypothesis that various kinds of sound exposures influence long-lasting sensitivity-changes of responses through stress effects on the auditory nerve level. We additionally have to consider that immediate GC-mediated activities operate within minutes to hours after exposure (de Kloet, 2014) preceding BDNF-mediated actions. Accordingly, dependence of BDNF on Arc adaptation processes is documented only for late but not early activities (Chowdhury et al., 2006; Nakayama et al., 2015; Carmichael and Henley, 2018; Epstein and Finkbeiner, 2018). Consequently, the activation of glutamate-receptors by injection of kainaic acid into BLEV mice increased activity-dependent *Bdnf* transcription within hours (Singer et al., submitted). As both effects occur during a similar timeframe, it is likely that sound exposure may promote the activation of *Bdnf* promoters IV and VI in the brainstem (Figure 2) following immediate corticosterone-induced accentuation of auditory fiber responses. The failure of *Bdnf* exon-IV-CFP and VI-YFP upregulation in the olfactory bulb after acoustic enrichment indicates that BDNF transcripts are not elevated ubiquitously but in an auditory-specific manner. While it is well established that activation of the *Bdnf* exon-IV promoter occurs in response to elevated calcium (Ca^2+^) levels subsequently to neuronal activity changes (West et al., 2014), it is only modestly activated by neuronal activity (Timmusk et al., 1993; Aid et al., 2007). It may, however, be activated indirectly in response to the same stimulus, through the involvement of the cAMP response element binding-protein (CREB) (Bambah-Mukku et al., 2014). Moreover, as previously proposed, *Bdnf* exon-IV in platelets may respond to the activity of store-operated calcium channels (Chacón-Fernández et al., 2016) and may thus be influenced by neuronal activity, which is known to tightly regulate blood flow (Hillman, 2014). Accordingly, a sound exposure-induced modification of auditory nerve activity may correspond to the driving force of altered activation of BDNF promoters in brainstem neurons, targeted by auditory nerves. In line with this, adaptations of *Bdnf* exon-IV-CFP and VI-YFP levels in the brainstem appear to correlate with increased expression of VGLUT1 (Figure 2), a specific presynaptic marker for auditory-specific synapses in the brainstem (Zhou et al., 2007). This indicates elevated numbers of active release sites, which were previously associated with greater spike fidelity of auditory specific synapses (Ngodup et al., 2015). Interestingly, BDNF regulates VGLUT1 expression during development and hippocampal LTP (Melo et al., 2013), and is able to prevent VGLUT1 reduction in cognitive diseases (Anglada-Huguet et al., 2016). This suggests that *Bdnf* exon-IV-CFP and VI-YFP levels in the brainstem following enriched or severe acoustic trauma may directly drive increased or reduced expression of VGLUT1, respectively. This process needs to be regarded in the context of activity-dependent BDNF which induces strengthening of synapses (Kellner et al., 2014) and acts as trans-synaptic messenger to control auditory-specific excitability.

### Increased activity in the auditory system enhances hippocampal BDNF transcription and synaptic plasticity

We also observed a trend for increased usage of *Bdnf* exon-IV and -VI in the hippocampus similar to the brainstem and accompanied by elevated levels of the AMPAR subunit GluA2, a marker for synaptic activity in the hippocampus (Tanaka et al., 2000), after acoustic enrichment, but not after severe acoustic trauma (Figure 3). Thus, we may assume that BDNF-modified auditory-specific excitability changes after enriching or traumatic sound exposure reach the hippocampus. Indeed, neuronal activity of the auditory pathway after sound exposure can propagate from auditory association cortices through the entorhinal cortex (EC) and via the performant path (PP) into the dorsal hippocampus (Munoz-Lopez et al., 2010). Activation of the dorsal hippocampus was shown after stress (Fanselow and Dong, 2010; Kirby et al., 2013) and environmental enrichment (Tanti et al., 2012), processes that also led to increased LTP (Korz and Frey, 2005) or to improved memory (Hullinger et al., 2015). Furthermore, like environmental enrichment (Weinberger, 2003; Chavez et al., 2009), acoustic enrichment improves frequency discrimination in the auditory cortex as well as the sensitivity to quiet sounds (Engineer et al., 2004; Cai et al., 2009; Bose et al., 2010; Centanni et al., 2013) by a mechanism that most likely involves projection pathways of the medial geniculate body (MGB) (Malmierca and Merchan, 2004). Fittingly, the present study shows acoustic enrichment to coincide with elevated LTP and improved performance in a hippocampus-dependent memory task (Figure 3). Moreover, these processes led to *Bdnf* exon-VI-YFP fluorescence that was most prominently increased in the hippocampal CA3 region, while B*dnf* exon-IV-CFP expression was highest in capillaries of the FH and in PC soma (Figure 4). Mossy fiber terminals in the CA3 area drive rapid generation and contextualization of episodic memories through elevated neurogenesis and feed-forward inhibition (Donato et al., 2013), for example in response to environmental enrichment. This process fails in post-traumatic stress disorders (Kheirbek et al., 2012; Zaletel et al., 2017). We thus may assume that unlike severe acoustic trauma, acoustic enrichment and mild acoustic trauma improve the generation and contextualization of memory traces because the exposure conditions modify auditory input in such a way that it activates *Bdnf* transcripts in the brainstem and alters auditory-specific ascending glutamatergic excitability. This constitutes an auditory-specific information flow that conveys to the hippocampus through the ascending auditory pathway. This assumption is supported by the failure to recruit *Bdnf* transcript activation in the brainstem and hippocampus following severe acoustic trauma that is also associated with a failure to restore sound sensitivity (ABR wave I and IV reduction, Figure 1).

In summary, the changes in *Bdnf* exon-IV usage in hippocampal projecting neurons and *Bdnf* exon-VI in mossy fibers may be interpreted as a result of a sound-induced alteration of the driving force that spreads along the auditory path from the cochlea in an auditory-specific manner. This model could explain the simultaneous changes in the expression of the excitatory marker GluA2 and BDNF in the hippocampus that interestingly coincided with the expression of PV, a marker for a wide array of inhibitory interneurons (Kepecs and Fishell, 2014). Particularly fast-spiking PV-positive interneurons serve a crucial function for microcircuit formation (Hu et al., 2014) and feed-forward inhibition (Donato et al., 2013) under control of BDNF (Waterhouse et al., 2012). PV-positive interneurons are also targeted by BDNF-expressing mossy-fibers and there is evidence that PV expressing basket-cell (BC) interneurons are activated by BDNF (Danzer and Mcnamara, 2004; Danzer et al., 2008). The changes in PV expression observed here could therefore be the direct result of excitability changes arising from the auditory input side. This would perfectly explain the all-encompassing correlation of events that differed between sound-exposure paradigms. In addition, altered PV and BDNF expression patterns observed after sound exposure share characteristics of feed-forward inhibition. First, there is a correlation of *Bdnf* exon-VI-YFP levels in mossy fibers and PV-positive neurons in the CA1 that are likely to correspond to BCs (Figure 6). BCs contact neighboring PCs through perisomatic δ subunit containing GABA_A_-receptor positive synapses (Klausberger et al., 2003; Glykys et al., 2008). Here we observed *Bdnf* exon-IV-CFP expressing PCs and PV-positive BCs that express δ subunit containing GABA_A_-receptors located nearby the PC soma (Figures 7C,F). Future studies might demonstrate recruitment of δ subunit containing GABA_A_ receptors to *Bdnf* exon-VI-YFP positive synapses in the CA1 pyramidal layer as previously observed (Glykys et al., 2008). Furthermore, we found a reduction of PV and α1 subunit containing GABA_A_-receptor IR in the SR of animals exposed to acoustic enrichment (80 dB SPL) or mild acoustic trauma (100 dB SPL, Figures 6C,E; Supplementary Figures 5D,F). This may represent BC-mediated inhibition of PV-positive bistratified (BS) interneurons that target CA1 dendrites through α1 subunit containing GABA_A_-receptor expressing synapses (Klausberger and Somogyi, 2008; Willadt et al., 2013). The present findings in BLEV mice do not support a causal link of BDNF levels with PV-mediated feed-forward inhibition. The absence of *Bdnf* transcript mobilization in the tri-synaptic path together with unaltered PV expression and LTP after the reduction of auditory input by severe acoustic trauma (120 dB SPL, Figures 7D–F), however, indicates that activity-dependent BDNF expression in the hippocampal tri-synaptic path is a consequence of task-specific BDNF activities in lower auditory brainstem regions. This might be the initial cue to adapt the processing of auditory information within the circuits. It is challenging to consider that during memory-linked adaptation processes the previously shown mossy cell-mediated BDNF-dependent enhancement of dentate granule cell output to the CA3 region (Hashimotodani et al., 2017) might also be activated. The monitoring of these processes is now empowered in BLEV mice.

Our findings thus provide a model for a general mechanism through which sound stimulation is linked to behaviorally relevant alterations in activity of neural networks. Furthermore, our data suggest that the precise nature of auditory experience may sculpt synaptic traffic in the auditory brain and its connections, including regions such as the hippocampus that are critical for navigating the environment and mapping memories. We propose that a mechanism of this kind may be relevant to establish critical auditory periods in different species including humans (Chen and Yuan, 2015) and may also play an important role in the pathophysiology of human neurodegenerative diseases. Hearing loss has been shown to be associated with an increased risk of dementia in epidemiological studies (Lin et al., 2017), however the nature of this linkage has not been defined. Loss of a critical driving force for recruitment of activity-dependent BDNF expression in neuronal, glial and vascular cells is a plausible mechanism that could contribute to a non-adapting metabolic supply. This may cause accelerated regional brain (in particular, parahippocampal) atrophy (Lin et al., 2014) followed by chronic peripheral hearing impairment such as in older people developing Alzheimer's disease. The findings suggest that possible neurodegenerative proteinopathies (Hardy et al., 2016) may not necessarily be overcome by systemic BDNF therapies. In contrast, sustained patterns of neural network activity may promote the spread of specific proteinopathies (“molecular nexopathies”) (Warren et al., 2013), and BDNF expression might be one mediator of such activity-dependent network degenerations in the setting of proteinopathies, as recently also demonstrated in Alzheimer's disease (Hardy et al., 2017). From the finding in the present study it is challenging to speculate that “auditory enrichment” (for example, via regular music listening) might also engage a BDNF-dependent mechanism, with intriguing implications for neuroprotective strategies.

In conclusion, here we demonstrate for the first time that translation of exon-IV and -VI derived BDNF is elevated after sound exposure conditions that induce long-lasting changes in sound-sensitivity correlating with increased hippocampal LTP. We verify the BLEV reporter mouse as a model to identify and examine those neurons, non-neuronal glial and capillary cells that in a task-specific and orchestrated way respond to those environmental changes that induce behavioral relevant adaptation processes. BLEV mice may thus be used to demonstrate that the synchronized activation of BDNF in neurons, glia, and capillaries provides the specific cues for GC-mediated metabolic support (de Kloet, 2014; Jeanneteau and Arango-Lievano, 2016) in the context of a specific sensory organ, a hypothesis that needs to be tested in more detail in future studies. To sum up, BLEV mice allow monitoring of which networks and which of their parts are activated and altered to accentuate behaviorally important auditory input (Berlau and Weinberger, 2008; Munoz-Lopez et al., 2010; Kraus and White-Schwoch, 2015). Our findings suggest a candidate mechanism whereby auditory experience may sculpt neural networks in the ascending auditory pathway and beyond. This in turn has potentially wide-reaching implications for understanding the role of auditory stimulation in promoting normal development of the human auditory brain and the contribution of auditory dysfunction to disease states, notably the neurodegenerative proteinopathies.

## Author contributions

WS, PE, LM, RP-W, H-SG, TO, and MK: Conceptualization. WS, LM, AB, PE, MM, CH, EF, LR, and MK: Analysis. WS, LM, H-SG, AB, PE, MM, NM, and KR: Investigation. WS, RP-W, LM, PE, UZ, LR, TS, and MK: Writing. WS, PR, EF, TO, LR, and MK: Supervision. LM, WS, PR, UZ, LR, and MK: Review and Editing.

### Conflict of interest statement

The authors declare that the research was conducted in the absence of any commercial or financial relationships that could be construed as a potential conflict of interest.
