# Supplementary material for: Visualizing BDNF Transcript Usage During Sound-Induced Memory Linked Plasticity
Source: Front Mol Neurosci. 2018 Jul 31;11:260. doi: 10.3389/fnmol.2018.00260 (PMC6089339; doi:10.3389/fnmol.2018.00260)
Supplement: Supplementary file 1 [file Data_Sheet_1.PDF]

Supplementary Figure 1

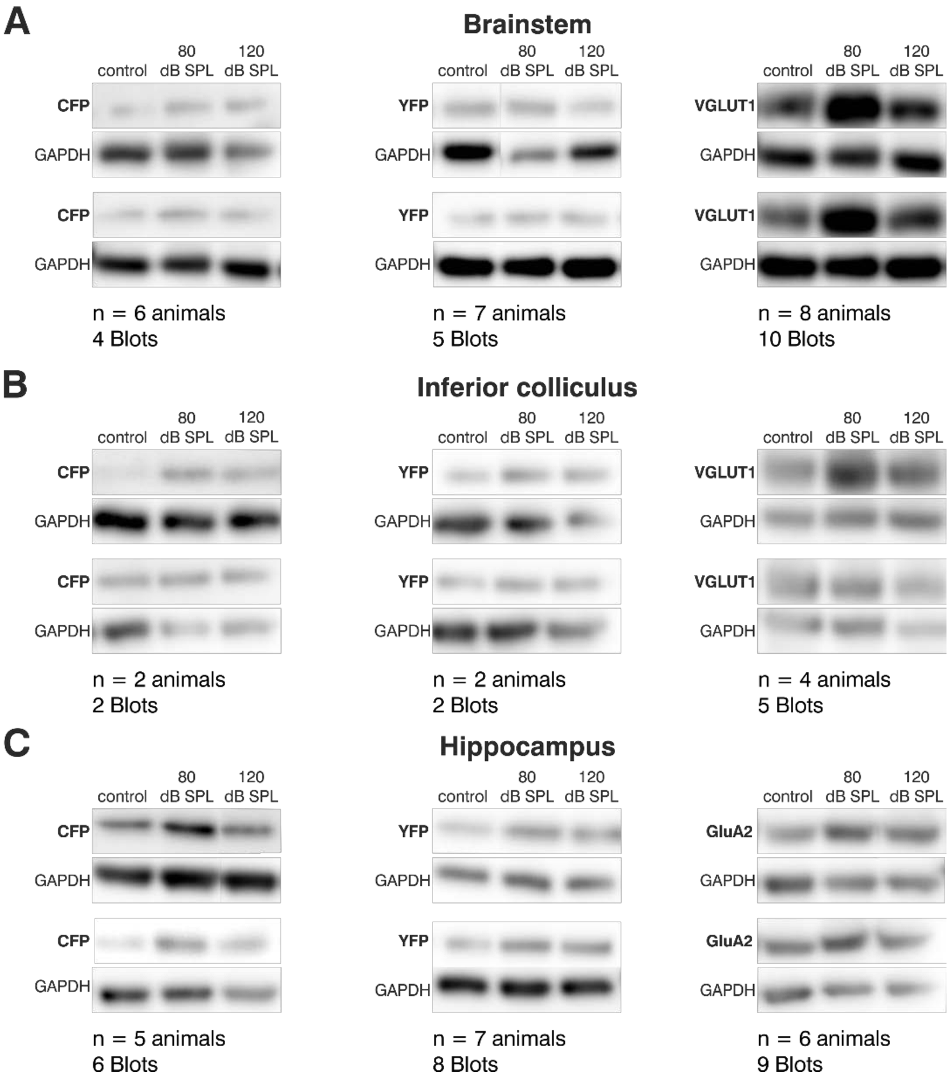


**Supplementary Figure 1.** Western blot analyses of CFP and YFP for (**A**) brainstem, (**B**) inferior colliculus and (**C**) hippocampus in control mice and mice exposed to 80 or 120 dB SPL. For brainstem and inferior colliculus, VGLUT1 was additionally analyzed, and for the hippocampus, GluA2 was additionally analyzed. Two examples are shown for each tissue and antibody. GAPDH is used as house-keeping gene. Note an increase of CFP, YFP, VGLUT1 and GluA2 in all tissues of mice exposed to 80 dB SPL. For originals see **Supplementary Figure 3**.

## Supplementary Figure 2

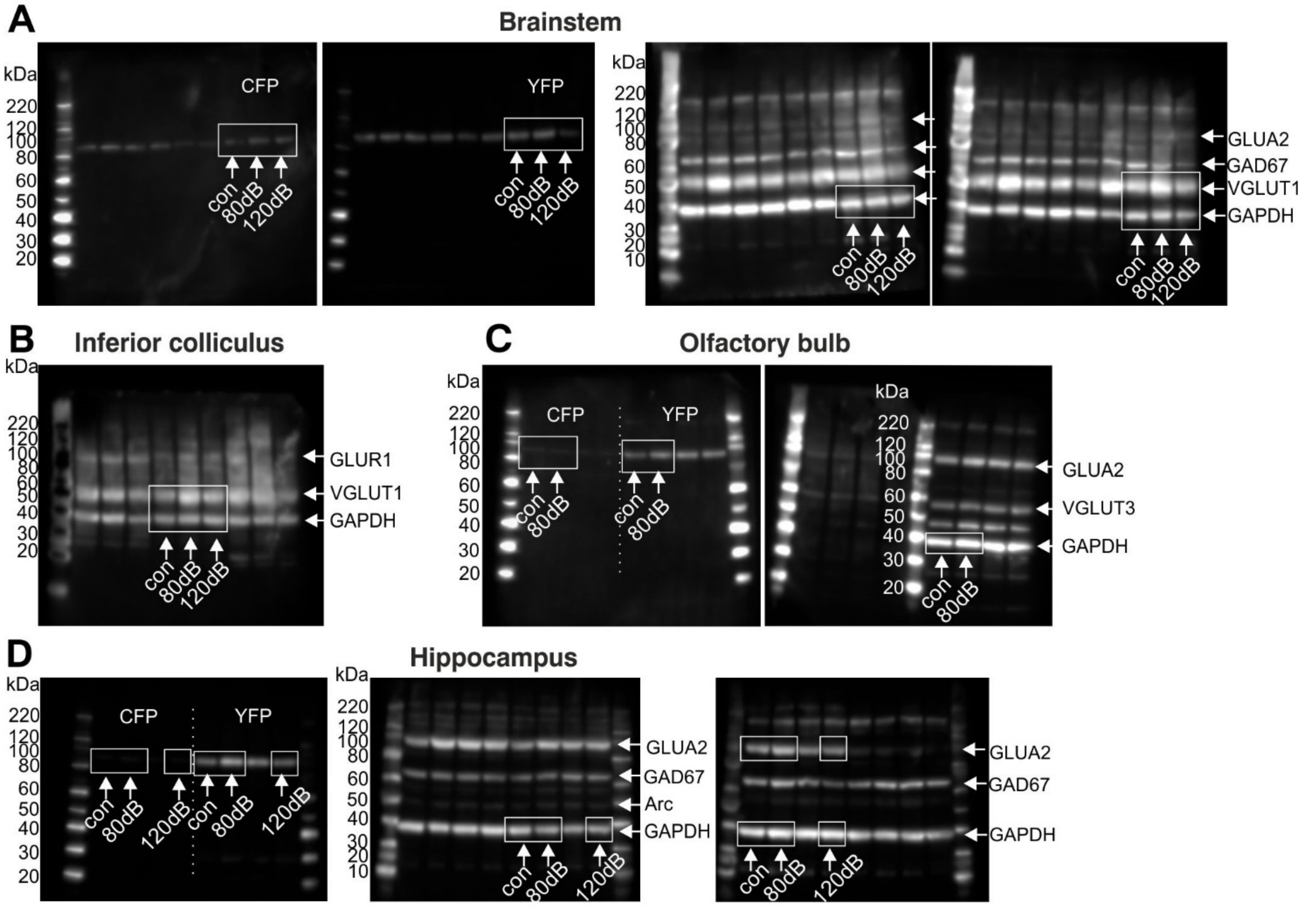


**Supplementary Figure 2.** Original Western blots for Figure 2 A (**A-C**) and Figure 3 A (**D**). (**A**) – (**D**) Arrows indicate bands used in Figures. Images were inverted and for better visibility the contrast was adjusted. All quantitative analyses were performed on original images (**A**) Original Western blots for CFP, YFP, vGlut1 and GAPDH in the brainstem. (**B**) Original Western blots for vGlut1 and GAPDH in the inferior colliculus. (**C**) Original Western bolts for CFP, YFP and GAPDH in the olfactory bulb. (**D**) Original Western blots for CFP, YFP, GluA2 and GAPDH in the hippocampus.

Supplementary Figure 3

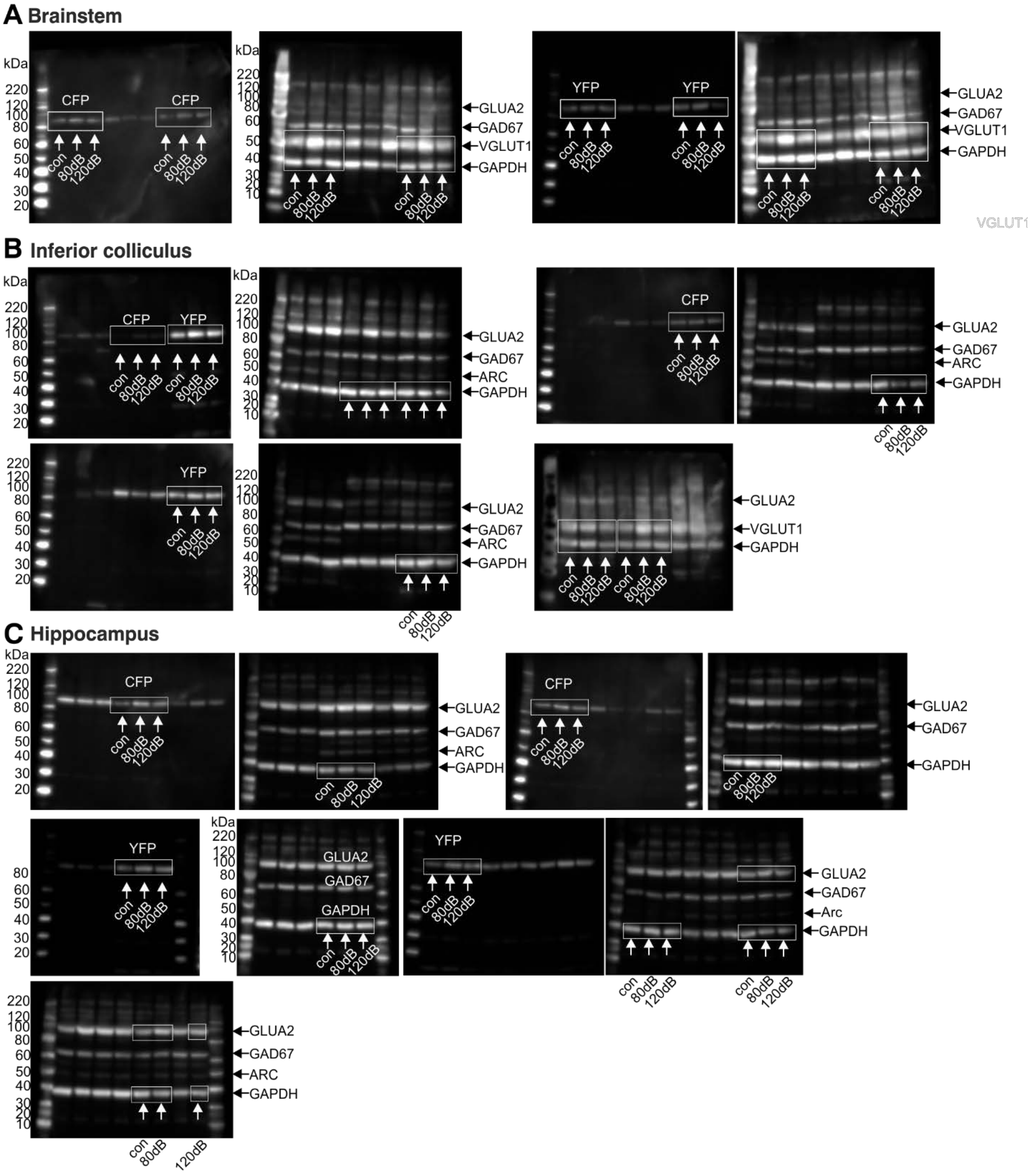


**Supplementary Figure 3.** Original Western blots for Supplementary Figure 1. Arrows indicate bands used in Supplementary Figure 1. Images were inverted and for better visibility the contrast was adjusted. All quantitative analyses were performed on original images (**A**) Original Western blots for CFP, YFP, vGlut1 and GAPDH in the brainstem. (**B**) Original Western blots for CFP, YFP, vGlut1 and GAPDH in the inferior colliculus. (**C**) Original Western blots for CFP, YFP, GluA2 and GAPDH in the hippocampus.

## Supplementary Figure 4

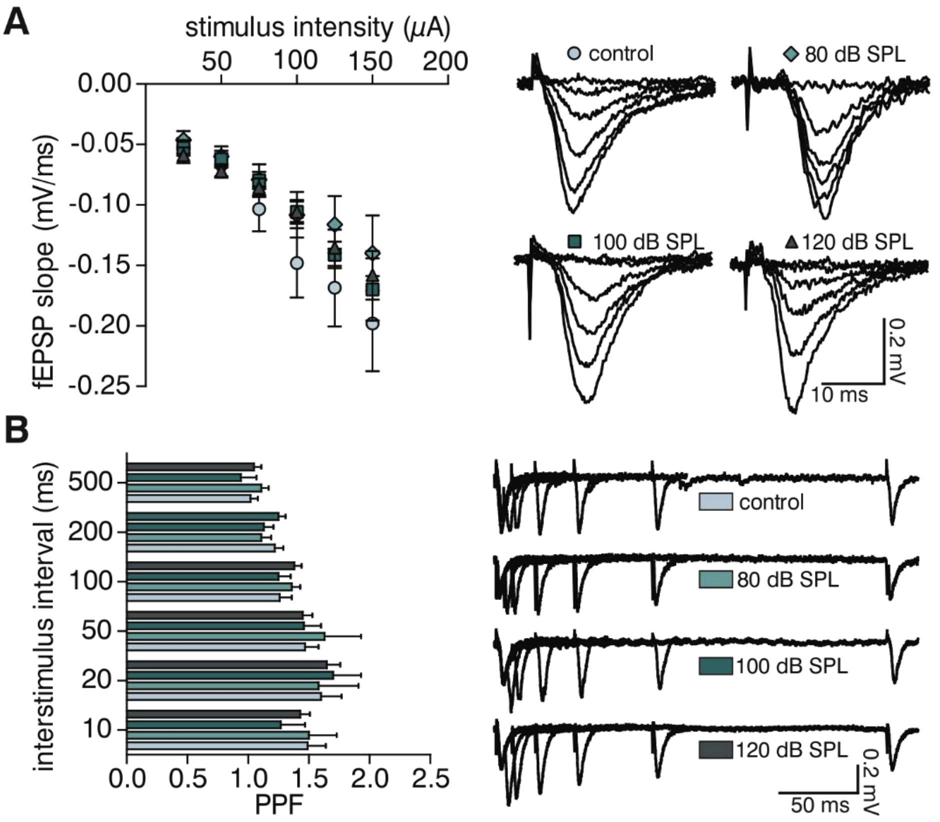


**Supplementary Figure 4.** (**A, B**) Schaffer collateral fEPSPs were recorded from acute forebrain slices of 8-12-week-old mice. (**A**) Average fEPSP slope was plotted against stimulus intensity. No difference was observed between slices from controls or animals exposed to 80 dB SPL, 100 dB SPL, and 120 dB SPL. Traces from representative recordings are shown on the right. Data represented as mean ± SEM (con $n = 11$ slices, 80 dB SPL $n = 14$ slices, 100 dB SPL $n = 11$ slices, 120 dB SPL $n = 14$ slices.) (**B**) Paired-pulse facilitation (PPF) was not different between slices from controls or animals exposed to 80 dB SPL, 100 dB SPL, and 120 dB SPL for all inter-stimulus intervals (I.S.I.). Traces from representative recordings are shown on the right. Data represented as mean ± SEM (con $n = 10$ slices, 80 dB SPL $n = 13$ slices, 100 dB SPL $n = 11$ slices, 120 dB SPL $n = 15$ slices).

Supplementary Figure 5

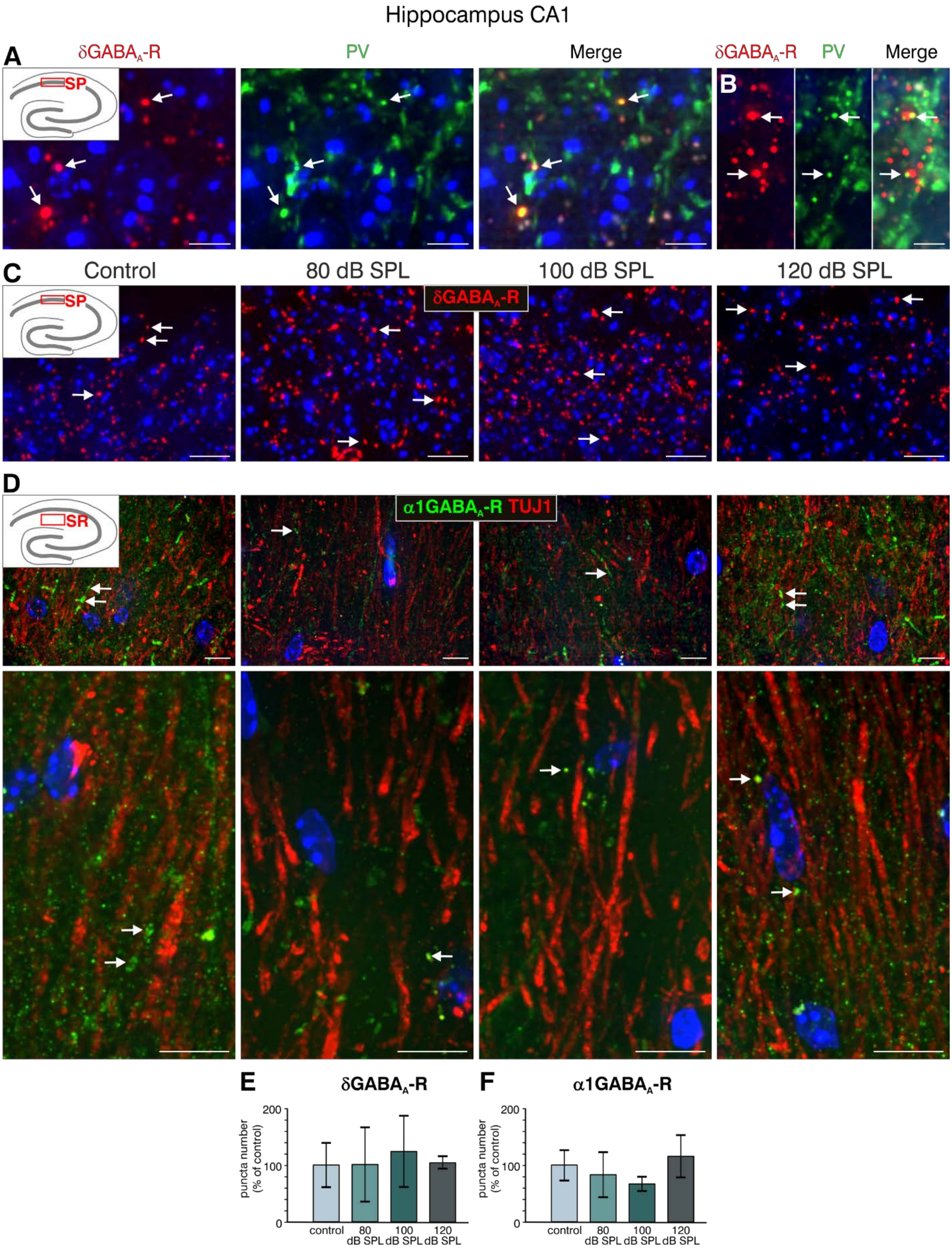


**Supplementary Figure 5.** Expression of δ subunit containing $GABA_A$ receptors (δ$GABA_A$-R) and α subunit containing $GABA_A$ receptor (α1$GABA_A$-R) in the hippocampal CA1 region. (**A-C**) Hippocampal CA1 region with magnification of the stratum pyramidale (SP) stained for δ$GABA_A$-R (red) and parvalbumin (PV, green). (**A**) Note the co-localization of PV with δ$GABA_A$-R-positive dots (arrows). Scale bars: 5 µm. (**B**) δ$GABA_A$-R-positive dots are contacted by PV-positive dendrites (arrows) of CA1 interneurons. Scale bars: 2.5 µm. (**C**) No obvious changes are observed in δ$GABA_A$-R expression (red, arrows) between the different treatment groups. Scale bars: 10 µm. (**D**) Hippocampal CA1 region with magnification of the stratum radiatum (SR)

stained for αGABA$_A$-R (green, arrows) and TUJ1 (red). Note the dynamic change in αGABA$_A$-R expression dependent on sound exposure (green, arrows). Bottom panel: High-power examination of the SR. Changes in αGABA$_A$-R expression around TUJ1-positive neurons can be observed. Scale bars: 10 µm. (*n* = 4-5 animals /group). (**E**) Quantification of δGABA$_A$-R (1-way ANOVA: F(3, 14) = 0.22 $p = 0.88$; con, 80 dB SPL $n = 5$ animals; 100 dB SPL, 120 dB SPL $n = 4$ animals) and (**F**) αGABA$_A$-R fluorescence in the CA1 region. Data represented as mean ± SD (1-way ANOVA: F(3, 16) = 2.08 $p = 0.14$; $n = 5$ animals / group 2-3 repetitions).

**Supplementary Table 1** Antibody information for immunohistochemistry and Western blot

| Primary Antibodies | | | | | | |
|---|---|---|---|---|---|---|
| Immunohistochemistry | Antibody | Protein name | Product number | Source | Dilution | Protein size |
| Cochlea / Brain sections | rabbit anti-CtBP2/ RIBEYE | CtBP2/RIBEY E | 10-P1554 | American research Products, Inc., | 1:1,000 | |
| | rabbit anti-parvalbumin | Parvalbumin | ab11427 | Abcam | 1:2,000 | |
| | mouse anti-parvalbumin | Parvalbumin | P3088 | Sigma-Aldrich | 1:500 | |
| | guinea pig anti-α1GABA$_A$-receptor | Gamma-aminobutyric acid type A receptor α subunit | 224204 | Synaptic Systems | 1:500 | |
| | rabbit anti-δGABA$_A$-receptor | Gamma-aminobutyric acid type A receptor δ subunit | AB9752 | Millipore | 1:1,500 | |
| | guinea pig anti-VGLUT1 | Vesicular glutamate transporter 1 | 135304 | Synaptic Systems | 1:1,500 | |
| Western blot | rabbit anti-RCFP | Reef coral fluorescent protein pan antibody | 632475 | Clontech | 1:1,000 | 30-35 kDa |
| | guinea pig anti-VGLUT1 | Vesicular glutamate transporter 1 | 135304 | Synaptic Systems | 1:5,000 | 50 kDa |
| | rabbit anti-GluA2 | Glutamate receptor AMPA 2 | 182103 | Synaptic Systems | 1:1,500 | 100 kDa |
| | mouse anti-GAPDH | Glyceraldehyd e 3-phosphate dehydrogenase | ab8245 | Abcam | 1:10,00 0 | 40 kDa |
| Secondary antibodies | | | | | | |
| Immunohistochemistry | Antibody | Protein name | Product number | Source | Dilution | Protein size |
| Cochlea / Brain sections | Cy3-conjugated goat anti-rabbit antibody | | 111-166-003 | Jackson ImmunoR esearch Laborator ies | 1:1,500 | |
| | Alexa488-conjugated anti-mouse antibody | | A11001 | Molecular Probes, MoBiTec | 1:500 | |
| | Alexa488-conjugated anti-guinea pig antibody | | A11073 | Molecular Probes, MoBiTec | 1:500 | |
| Western blot | ECL anti-mouse IgG HRP linked | | NA 931-100µl | GE Healthcar | 1:2,500 | |

| | | | | | | |
|---|---|---|---|---|---|---|
| | | | | e UK Limited | | |
| | ECL anti-rabbit IgG HRP linked | | NA 934-100µl | GE Healthcare UK Limited | 1:2,500 | |
| | Goat anti-gionea pig IgG-HRP | | Sc-2438 | Santa Cruz Biotechnology Inc. | 1:7,000 | |

**Supplementary Table 2** Statistical information of the results

| | | **Statistical test** | **Test value** | **Degrees of freedom** | ***p*-value** | **Post-hoc test with *p*-value** | | **n-number** |
|---|---|---|---|---|---|---|---|---|
| Fig. 1A | Click-ABR TTS post AT | 1-way ANOVA | F(3, 129) = 92.67 | | p < 0.0001 | Bonferroni's test<br>con vs. 80 dB SPL<br>con vs. 100 dB SPL<br>con vs. 120 dB SPL | p<0.0001 | con n = 19 animals<br>80 dB SPL n= 19 animals<br>100 dB SPL n = 16 animals<br>120 dB SPL n = 19 animals |
| | Click-ABR 14d post AT | 1-way ANOVA | F(3, 144) = 54.72 | | p < 0.0001 | con vs. 120 dB SPL | p <0.0001 | |
| Fig. 1B | noise-ABR 14d post AT | 1-way ANOVA | F(3, 142) = 75.45 | | p < 0.0001 | con vs. 120 dB SPL | p < 0.0001 | |
| Fig. 1C | f-ABR | 2-way ANOVA | F(3, 597) = 79.9 | | p < 0.0001 | con vs. 120 dB SPL | p < 0.05 | |
| Fig. 1E | ribbon number | 2-way ANOVA | F(3, 60) = 11.08 | | p < 0.0001 | 1-sided unpaired Student's t-tests<br>middle turn: con vs. 80 dB SPL<br>con vs. 120 dB SPL<br>midbasal turn: con vs. 100 dB SPL<br>con vs. 120 dB SPL | p < 0.05<br>p < 0.01<br>p < 0.05<br>p < 0.01 | n = 6 ears from 4 animals per group, 1-3 repetitions each, 8-24 IHCs per turn and group |
| Fig. 1F | CorF | 1-way ANOVA | F(3, 131) = 17.51 | | p < 0.0001 | Tukey's Multiple Comparison test:<br>con vs. 120 dB SPL<br>80 dB SPL vs. 120 dB SPL<br>100 dB SPL vs. 120 dB SPL | p < 0.001<br>p < 0.001<br>p < 0.01 | con n = 8 animals, 15 ears;<br>80 dB SPL n = 9 animals, 18 ears<br>100 dB SPL n = 5 animals, 10 ears;<br>120 dB SPL n = 9 animals, 17 ears |
| Fig. 1G | wave I control | 2-way ANOVA | F(1, 1031) = 0.003 | | p = 0.955 | | | n = 18 mice/group |
| | wave I 80 dB SPL | 2-way ANOVA | F(1, 890) = 6.02 | | p = 0.0143 | | | n = 16 mice/group |
| | wave I 100 dB SPL | 2-way ANOVA | F(1, 836) = 28.59 | | p < 0.0001 | | | n = 15 mice/group |

| | | | | | | | | |
|---|---|---|---|---|---|---|---|---|
| | wave I<br>120 dB<br>SPL | 2-way ANOVA | F(1, 396) =<br>185.8 | | p < 0.0001 | | | n = 18 mice/group |
| Fig.<br>1H | wave I V<br>control | 2-way ANOVA | F(1, 1034) =<br>1.296 | | p = 0.2551 | | | n = 18 mice/group |
| | wave I V<br>80 dB<br>SPL | 2-way ANOVA | F(1, 951) = 0.89 | | p = 0.3446 | | | n = 16 mice/group |
| | wave I V<br>100 dB<br>SPL | 2-way ANOVA | F(1, 743) = 0.09 | | p = 0.7706 | | | n = 15 mice/group |
| | wave I V<br>120 dB<br>SPL | 2-way ANOVA | F(1, 452) =<br>82.88 | | p < 0.0001 | | | n = 18 mice/group |
| Fig.<br>2A/B | | | | | | | | n = 5 - 6 mice/group<br>2 - 10 Western blots<br>each |
| Fig.<br>2C/D | VGLUT1 | two-tailed<br>student's t-test | t = 3.63<br>df = 10 | | p = 0.0046 | | | n = 6 mice/group<br>2 - 3 repetitions |
| Fig.<br>3A | | | | | | | | n = 5 mice/group<br>2 - 10 Western blots<br>each |
| Fig.<br>3B | fEPSP | 1-way ANOVA | F(3, 19) = 4.99 | | p = 0.01 | Bonferroni’s test<br>baseline/tetanized<br>(b/t) | | n = 4 animals /group<br>con 7 slices<br>80 dB SPL 7 slices<br>100 dB SPL 6 slices<br>120 dB SPL 5 slices |
| | | | | | | control | p < 0.01 | |
| | | | | | | 80 dB SPL | p < 0.001 | |
| | | | | | | 100 dB SPL | p < 0.001 | |
| | | | | | | 120 dB SPL | p < 0.01 | |
| | | | | | | tetanized/tetanized<br>(t/t) | | |
| | | | | | | con vs. 80 dB SPL | p < 0.01 | |
| | | | | | | con vs. 100 dB<br>SPL | p< 0.01 | |
| | | | | | | con vs. 120 dB<br>SPL | n.s. | |
| Fig.<br>3C | con | repteaed measure<br>ANOVA | F = 2.56 | DF = 5 | p = 0.033 | Bonferroni’s test | | con n= 18 animals<br>80 dB SPL n = 17<br>animals |
| | | | | | | day 1 vs. day 2 | | |
| | | | | | | day 1 vs. day 3 | | |
| | | | | | | day 1 vs. day 4 | n.s. | |
| | | | | | | day 1 vs. day 5 | | |
| | | | | | | day 1 vs. day 6 | | |
| | 80dB<br>SPL | | F = 5.85 | DF = 5 | p < 0.001 | day 1 vs. day 2 | n.s. | |
| | | | | | | day 1 vs. day 3 | p = 0.013 | |
| | | | | | | day 1 vs. day 4 | p = 0.054 | |
| | | | | | | day 1 vs. day 5 | p = 0.006 | |
| | | | | | | day 1 vs. day 6 | p < 0.001 | |

| | | | | | | | |
|---|---|---|---|---|---|---|---|
| Fig. 4A-C | | | | | | | n = 6 animals/group |
| Fig. 4D | CFP | 1-way ANOVA | F(3, 19) = 10.5 | p < 0.0003 | Bonferroni's test | | con n= 6 animals; 1-4 repetitions<br>80 dB SPL n = 6 animals; 1-6 repetitions<br>100 dB SPL n = 5 animals; 2-5 repetitions<br>120 dB SPL n = 6 animals; 1-3 repetitions |
| | | | | | con vs. 80 dB SPL | p = 0.0013 | |
| | | | | | con vs. 100 dB SPL | n.s. | |
| | | | | | con vs. 120 dB SPL | n.s. | |
| | | | | | 80 dB SPL vs. 120 dB SPL | p < 0.0001 | |
| | | | | | 100 dB SPL vs. 120 dB SPL | p = 0.0072 | |
| Fig. 4E | | | | | Bonferroni's test | | con n = 4 animals<br>80 dB SPL n = 3 animals<br>100 dB SPL n = 3 animals<br>120 dB SPL n = 5 animals;<br>3 repetitions each |
| | YFP | 1-way ANOVA | F(3, 11) = 6.96 | p = 0.0068 | con vs. 80 dB SPL | p < 0.01 | |
| | | | | | 80 dB SPL vs. 120 dB SPL | p < 0.01 | |
| | CFP | 1-way ANOVA | F(3, 11) = 2.37 | p = 0.13 | | | |
| | PV | 1-way ANOVA | F(3, 11) = 10.07 | p = 0.0017 | con vs. 80 dB SPL | p < 0.01 | |
| | | | | | 80 dB SPL vs. 120 dB SPL | p < 0.01 | |
| Fig. 5A-C | | | | | | | n = 6 animals/group |
| Fig. 5D | | | | | Bonferroni's test | | n=6 animals / group<br>4-6 repetition each |
| | CFP | 1-way ANOVA | F(3,25) = 22.44 | p < 0.0001 | con vs. 80 dB SPL | p < 0.001 | |
| | | | | | 80dB SPL vs. 120 dB SPL | p < 0.0001 | |
| | YFP | 1-way ANOVA | F(3, 25) = 3.32 | p = 0.036 | 80 dB SPL vs. 120 dB SPL | p < 0.05 | |
| Fig. 6A-C | | | | | | | n = 6 animals/group |
| Fig. 6D | | 1-way ANOVA | F(3, 19) = 5.96 | p = 0.0049 | Bonferroni's test | | con n= 6 animals<br>80 dB SPL n = 6 animals<br>100 dB SPL n = 5 animals<br>120 dB SPL n = 6 animals;<br>4-6 repetitions |
| | | | | | con vs. 80 dB SPL | n.s. | |
| | | | | | con vs. 100 dB SPL | p = 0.0301 | |
| | | | | | con vs. 120 dB SPL | n.s. | |
| | | | | | 100 vs. 120 dB SPL | p = 0.01 | |
| Fig. 6E | | 1-way ANOVA | F(3, 19) = 4.61 | p = 0.0138 | Bonferroni's test | | con n = 6 animals<br>80 dB SPL n = 5 animals<br>100 dB SPL n = 6 animals<br>120 dB SPL n= 6 animals<br>2 repetitions each |
| | | | | | con vs. 80 dB SPL | p < 0.05 | |
| | | | | | con vs. 100 dB SPL | p < 0.05 | |
| Fig. 6G | | 1-way ANOVA | F(3, 19) = 5.24 | p = 0.005 | Bonferroni's test baseline/wash-in | | n = 4 animals / group;<br>9 slices / group |
| | | | | | con | p < 0.001 | |
| | | | | | 80 dB SPL | n.s. | |
| | | | | | 100 dB SPL | n.s. | |

| | |
|---|---|
| 120 dB SPL | p < 0.001 |
| con vs. 120 dB SPL | p < 0.01 |
| con vs. 80 dB SPL | p < 0.01 |
| con vs. 100 dB SPL | n.s. |

**Supplementary Table 3** Statistical information of the supplementray information

| | | **Statistical test** | **Test value** | **Degrees of freedom** | ***p*-value** | **Post-hoc test with *p*-value** | **n-number** |
|---|---|---|---|---|---|---|---|
| S Fig. 4B | | | | | | | con n = 10 slices<br>80 dB SPL n = 13 slices<br>100 dB SPL n = 11 slices<br>120 dB SPL n = 15 slices |
| SFig. 5A-D | | | | | | | n = 4-5 animals /group |
| S Fig. 5E | δGABAA-R | 1-way ANOVA | F(3, 14) = 0.22 | | p = 0.88 | | con; 80 dB SPL n = 5 animals<br>100 dB SPL; 120 dB SPL n = 4 animals |
| S Fig. 5F | α1GABAA-R | 1-way ANOVA | F(3, 16) = 2.08 | | p = 0.14 | | n = 5 animals / group<br>2-3 repetitions |
